# ZmARF1 positively regulates low phosphorus stress tolerance via modulating lateral root development in maize

**DOI:** 10.1371/journal.pgen.1011135

**Published:** 2024-02-05

**Authors:** Fengkai Wu, Baba Salifu Yahaya, Ying Gong, Bing He, Junlin Gou, Yafeng He, Jing Li, Yan Kang, Jie Xu, Qingjun Wang, Xuanjun Feng, Qi Tang, Yaxi Liu, Yanli Lu

**Affiliations:** 1 State Key Laboratory of Crop Gene Exploration and Utilization in Southwest China, Sichuan Agricultural University, China; 2 Maize Research Institute, Sichuan Agricultural University, Wenjiang, Sichuan, China; 3 Key Laboratory of Biology and Genetic Improvement of Maize in Southwest Region, Ministry of Agriculture, China; 4 Triticeae Research Institute, Sichuan Agricultural University, Wenjiang, Sichuan, China; Peking University, CHINA

## Abstract

Phosphorus (P) deficiency is one of the most critical factors for plant growth and productivity, including its inhibition of lateral root initiation. Auxin response factors (ARFs) play crucial roles in root development via auxin signaling mediated by genetic pathways. In this study, we found that the transcription factor *ZmARF1* was associated with low inorganic phosphate (Pi) stress-related traits in maize. This superior root morphology and greater phosphate stress tolerance could be ascribed to the overexpression of *ZmARF1*. The knock out mutant *zmarf1* had shorter primary roots, fewer root tip number, and lower root volume and surface area. Transcriptomic data indicate that *ZmLBD1*, a direct downstream target gene, is involved in lateral root development, which enhances phosphate starvation tolerance. A transcriptional activation assay revealed that *ZmARF1* specifically binds to the GC-box motif in the promoter of *ZmLBD1* and activates its expression. Moreover, *ZmARF1* positively regulates the expression of *ZmPHR1*, *ZmPHT1;2*, and *ZmPHO2*, which are key transporters of Pi in maize. We propose that *ZmARF1* promotes the transcription of *ZmLBD1* to modulate lateral root development and Pi-starvation induced (*PSI*) genes to regulate phosphate mobilization and homeostasis under phosphorus starvation. In addition, ZmERF2 specifically binds to the ABRE motif of the promoter of *ZmARF1* and represses its expression. Collectively, the findings of this study revealed that *ZmARF1* is a pivotal factor that modulates root development and confers low-Pi stress tolerance through the transcriptional regulation of the biological function of *ZmLBD1* and the expression of key Pi transport proteins.

## Author summary

Phosphorus (P) is a critical macronutrient for plant growth and development. Root system architecture (RSA) affects the ability of plants to obtain water and mineral nutrients from soil. Several auxin response factors (ARFs) are involved in auxin signaling and lateral root (LR) formation in P-deficient plants, but the molecular mechanisms underlying P deficiency-induced changes in the RSA of maize is not well understood. We found that *ZmARF1* positively regulates root development and confers tolerance to low inorganic phosphate (Pi) stress in maize. *ZmARF1* regulates root development by interacting with the promoter of *ZmLBD1* and positively regulating its expression. Furthermore, we empirically revealed that *ZmARF1* enhanced Pi mobilization under low Pi stress by positively regulating the expression of *ZmPHR1*, *ZmPHT1;2*, and *ZmPHO2*, which are key Pi transporters in the Pi transport machinery of maize. Overall, enhanced root development and expression of key Pi transporter proteins have been suggested as key nexuses mediating the biological function of *ZmARF1* in conferring low-Pi stress tolerance in maize.

## 1. Introduction

Maize is a sessile plant that is constantly met with a myriad of biotic and abiotic stresses. Phosphorus (P) is an essential macronutrient that influences the growth and development of higher plants [1,2]. P is readily absorbed from the soil as inorganic phosphate (Pi), which is a primary limiting factor for optimal plant growth and development, overall yield, and quality [3]. P is an essential nutrient for agricultural ecosystems. Plants have developed intricate adaptive strategies to optimize growth and development under low-Pi conditions, including the modification of root morphology [4], activation of Pi-starvation induced (PSI) gene expression [5,6], and reprogramming of metabolic pathways [7].

The root system architecture (RSA) connotes the temporal and spatial distribution of roots within the soil and the growth rate of individual roots [8]. RSA plays crucial roles in an array of processes during the plant’s life cycle, including anchorage and nutrient and water absorption [9,10]. Although the regulation of root growth is highly complex, there is a general scientific consensus that root branching is genetically controlled and strongly influenced by biotic and abiotic environmental cues [8,11]. The RSA is highly plastic and enables plants to forage for increased soil volumes in Pi-rich patches under low Pi stress. The modification of root architecture in response to Pi limitation is an adaptive mechanism of crops to optimize their growth and productivity [12]. Low Pi stress in various plant species induces an increase in the root-to-shoot ratio, resulting from increased carbon redistribution from shoots to roots [13], increased root surface area [14], and the formation of longer root hairs [15]. Direct Pi uptake by plant roots involves Pi transporters, such as PHOSPHATE STARVATION RESPONSES (PHRs), high-affinity PHOSPHATE TRANSPORTERS (PHTs), and PHOSPHATE TRANSPORTER1 (PHO1) [5]. PHT proteins are actively involved in Pi uptake from the soil by roots and Pi redistribution in plants [16,17]. The PHO1 family of Pi transporters facilitates Pi loading from root epidermal and cortical cells into xylem vessels for long-distance Pi transportation from the root to shoot [18,19]. Transcription factors of the PHR1 family and their homologs form the central regulatory machinery that modulates the transcription of certain Pi-stress-responsive genes [20].

The auxin response factor (ARF) transcription factor (TF) family is one of the most important plant-specific modular TF families [21]. ARF proteins are crucial components of auxin transport and signaling cascades that play vital roles in lateral root development [22]. Members of the ARF family bind to the auxin response elements (AuxREs; TGTCTC) in the promoter regions of their target genes and regulate their expression [23]. ARF TFs have been implicated in numerous growth and developmental processes, as well as in the response to biotic and abiotic stresses. For example, *OsARF11* functions in the auxin-mediated growth of multiple organs and leaf veins and plays a central role in the formation of lateral roots, panicle branches, and grain meristems in rice [24]. *MtARF2*, *MtARF3*, and *MtARF4* mediate lateral root and nitrogen-fixing nodule development in *Medicago truncatula* [25]. *TaSAUR75* overexpression in Arabidopsis increases tolerance to drought and salt stress [26]. *ZmARF4* overexpression enhances lateral root growth in transgenic Arabidopsis and Pi mobilization by regulating *AtANS1* [27]. ARF7 and ARF19 regulate *PHR1* expression to mediate increased Pi uptake in roots and reduce anthocyanin accumulation in shoots under low Pi stress [28]. Transcriptome profiling has revealed that some members of the lateral organ boundary domain (LBD) TF family are downstream targets of ARFs involved in lateral root formation in auxin signaling pathways [29]. LBD TFs harbor an N-terminal lateral organ boundary (LOB) domain and play a crucial role in regulating organ development in plants, among which lateral root development is prominent [30,31]. For example, *MdARF26* and *MdARF27* upregulate the expression of *MdLBD16* to increase the length and number of adventitious roots under low Pi stress [32].

In this study, we isolated the maize Auxin Response Factor (*ZmARF1*) and characterized its function in root development and response to low Pi stress in maize. We identified significant associations between polymorphisms in *ZmARF1* and P tolerance traits in maize. *ZmARF1* expression was induced by Pi limitation. Overexpression of *ZmARF1* conferred low Pi tolerance to maize, whereas knockout of *ZmARF1* attenuated tolerance to low Pi stress. We found that *ZmLBD1* is a downstream target of *ZmARF1*, and its expression is upregulated by *ZmARF1* to promote root development. Simultaneously, *ZmERF2* binds to the promoter of *ZmARF1* and represses its transcription. Moreover, the expression levels of Pi transporters—*ZmPHR1*, *ZmPHT1;2*, and *ZmPHO2*—were significantly upregulated in *ZmARF1* overexpression maize lines, which consequently led to an increase in root and leaf Pi content, whereas the opposite was observed in the knockout mutant lines of maize.

## 2. Results

### 2.1 *ZmARF1* is associated with P stress tolerance in maize

**Fig 1:**
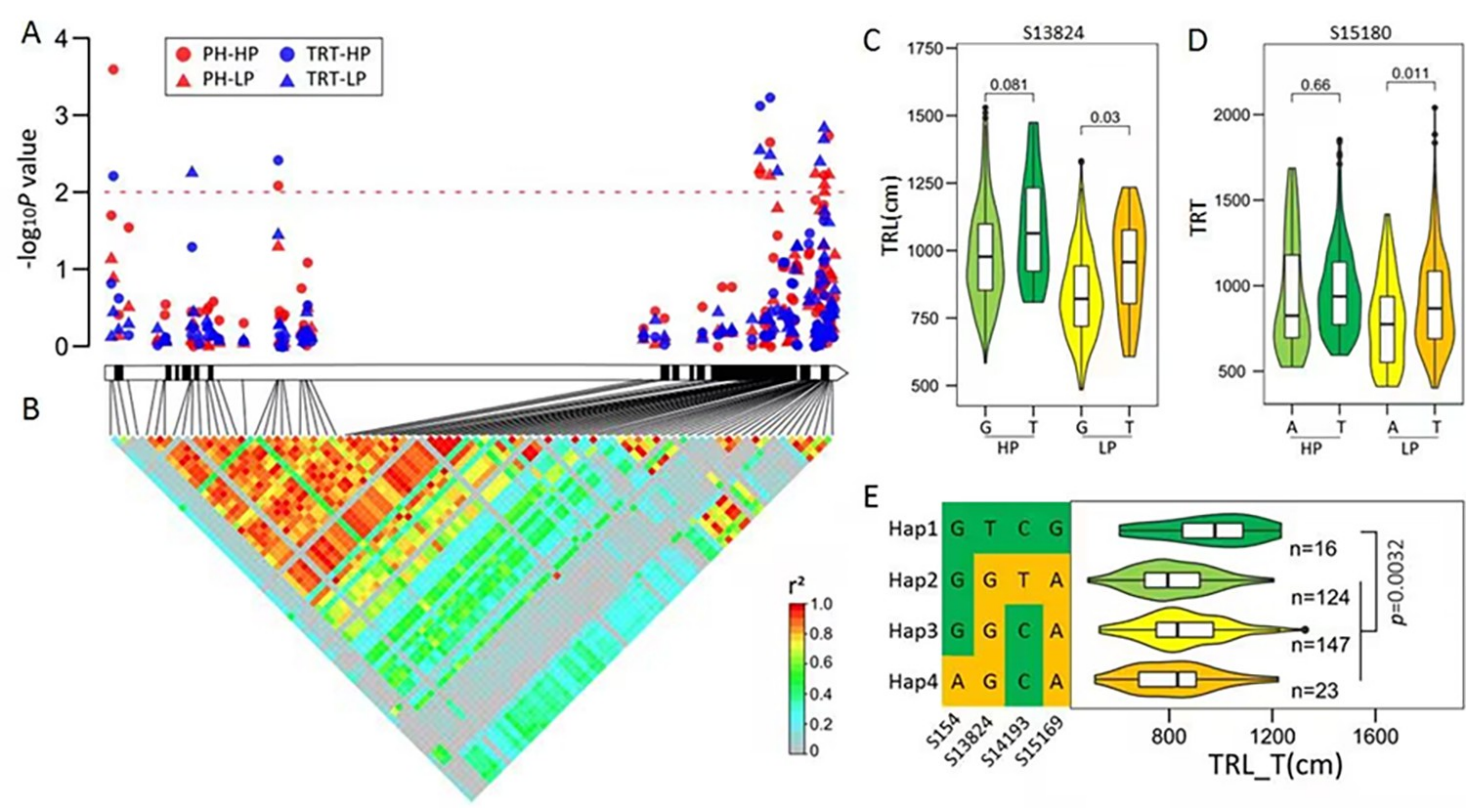
*ZmARF1*-based LD and association analysis in 356 diverse maize inbred lines. (A) Each circle or triangle represents an SNP site. The *P* value is shown on a -log10 scale. A schematic diagram of the entire gene structure of *ZmARF1* is presented as the x-axis, including white and black boxes representing noncoding regions and exons, respectively. HP and LP meaning normal condition in high-Pi and low-Pi stresses, respectively. (B) The pattern of pairwise LD of identified SNP in *ZmARF1*. The color reflects the level of LD (r^2^), and all polymorphic sites (MAF ≥ 0.05) are used. (C-D) Verification of excellent alleles significantly associated with TRL (C) and TRT (D) respectively. (E) Haplotypes of *ZmARF1* identified in the natural variation panel. n denotes the number of germplasms in each haplotype group. Statistical significance was determined by two-sided *t*-test.

To identify the natural variations in *ZmARF1* associated with the low-P stress response in maize, we performed a gene-based association analysis in 356 maize inbred lines. A total of 81 SNPs were identified in the full length of *ZmARF1* DNA segment with minor allele frequency ≥0.05 (S2 Table). The average SNP frequency among the 81 detected SNPs was one SNP per 191 bp (S2 Table). Two of the identified SNPs (S13824 and S15180) were significantly associated with total root length (TRL) and total root tip (TRT) number under low Pi (LP) conditions in all three tested models (Figs 1C, 1D, and S1 and S2 Table). SNP (S13824) was also significantly associated with plant height (PH) (Figs 1A and S1 and S2 Table), total root surface area (TRSA), and root fork (RF) (S2 Table) in at least two of the tested models. Moreover, 11 and 9 sites were significantly associated with TRT number and plant height (PH), respectively (S3 Table and Fig 1A). Overall, phenotypic variance explained a maximum of 3.756% of the variance. There were 29 loci in the intron, most of which were completely linked to SNP in the exon and formed large linkage disequilibrium (LD) blocks (Fig 1B).

Based on the 30 significant SNPs, we categorized the sequences of the 356 inbred maize lines into four haplotypes (Hap) (Fig 1E). Inbred lines carrying Hap1, represented by P178 (which has low Pi tolerance), exhibited the highest TRL after the low-Pi treatment. The germplasm carrying Hap4, represented by the low-Pi-sensitive line, 9782, exhibited the lowest TRL under low-Pi conditions. The haplotypes of the main plant developmental traits, such as TRT number, TRL, and PH, were highly consistent under both high Pi (HP) and LP conditions (S2 Fig). These results suggested that *ZmARF1* responds to Pi stress and simultaneously participates in the regulation of root and shoot development.

### 2.2 Characteristics of *ZmARF1* expression in response to low Pi stress

**Fig 2:**
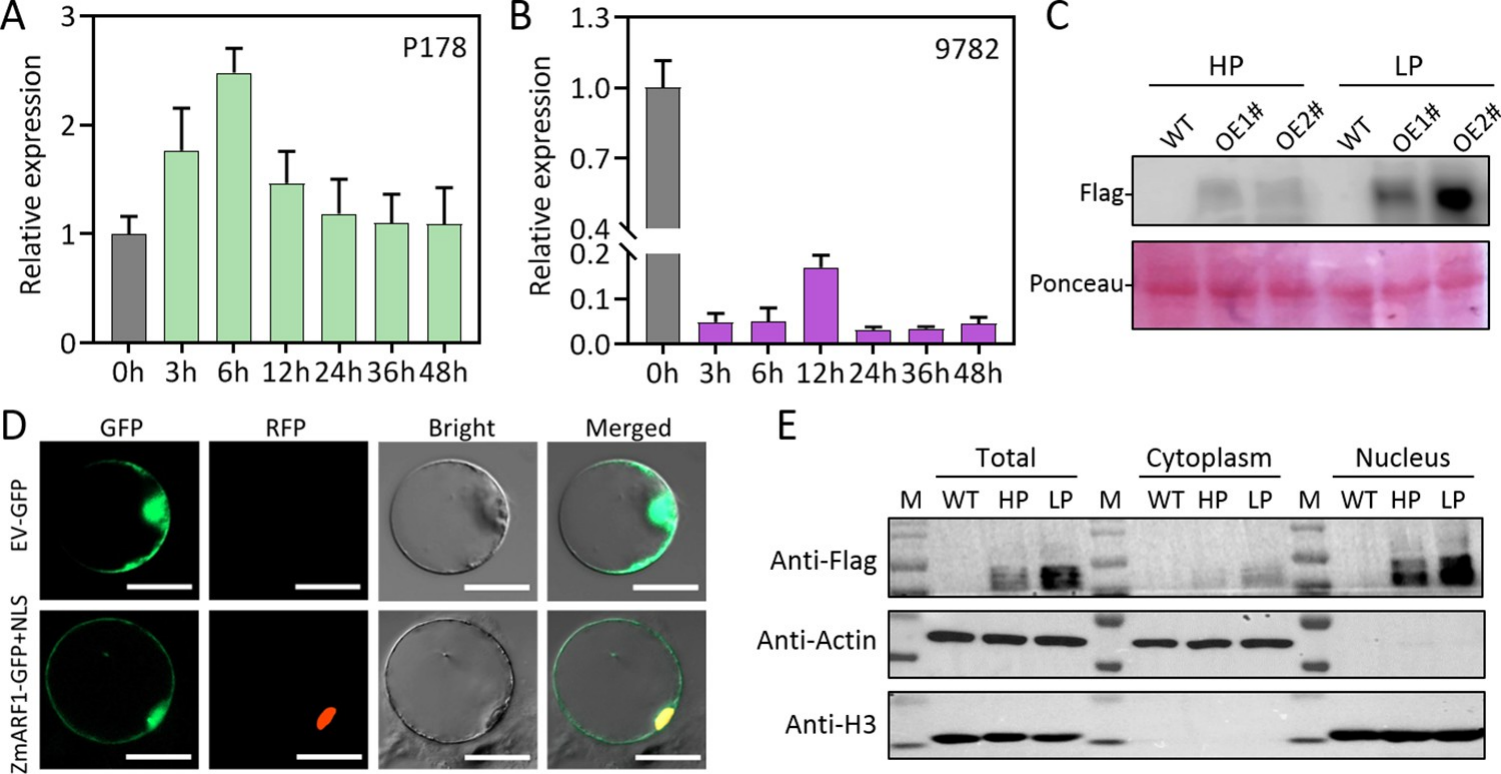
Expression characteristics of ZmARF1. Relative expression of ZmARF1 in roots of low-Pi tolerant (A) and low-Pi sensitive (B) maize inbred lines after low-Pi treatment. (C) Phosphate stress increased ZmARF1 protein abundance in maize. Two-week-old maize overexpression lines of ZmARF1 were subjected to high Pi (HP) and low Pi (LP) treatment for 14 days and ZmARF1 protein levels were revealed by immunoblotting using anti-Flag antibody. The experiment was repeated three times with the similar results. (D) Subcellular localization of ZmARF1-GFP fusion protein in maize protoplasts. The top and down panel represent CaMV35S::GFP and CaMV35S::ZmARF1-GFP expression, respectively. The extreme left is the GFP fluorescence, next to the right is the RFP nuclear localization signal of the nuclear marker, followed by the bright field signal and to the extreme right an overlap of the three images. Bar = 50 μm. (E) The abundance of ZmARF1 in total cell extracts, cytoplasmic, or nuclear fractions of roots from both WT and OE2# were assessed under HP and after 48 hours of LP treatment. The abundance of ZmARF1-3×Flag was determined through western blot using an anti-Flag antibody. Simultaneously, the abundance of cytoplasmic and nuclear fractions was determined using anti-Actin and anti-Histone (H3) as internal control, respectively.

The expression patterns of *ZmARF1* in the roots of Pi-tolerant (P178) and Pi-sensitive (9782) inbred lines were determined by RT-qPCR after low-Pi treatment. Low Pi treatment induced the expression of *ZmARF1* in P178; the expression of *ZmARF1* increased after 3 h of Pi induction and peaked at 6 h of treatment before decreasing to stable levels between 12 and 48 h after treatment (Fig 2A). In contrast, the expression level of *ZmARF1* in the low-Pi-sensitive inbred line decreased significantly after 3 h of low Pi treatment and remained constant until 48 h, with a maximum reduction of more 20 folds after 24 h of treatment (Fig 2B). The responsiveness of *ZmARF1* to low-Pi stress was further revealed by immunoblotting with an anti-Flag antibody. The results revealed that the expression of *ZmARF1* was significantly induced by low Pi stress, which was evident from an increase in *ZmARF1* protein abundance in the transgenic lines under low-Pi conditions relative to the high-Pi treatment (Fig 2C). This suggests that *ZmARF1* plays a significant regulatory role in the Pi stress response of maize.

To determine the subcellular localization of *ZmARF1*, the full coding region of *ZmARF1* without the stop codon was amplified and fused to the HBT95-GFP vector driven by the *CaMV35S* promoter. The CaMV35S::ZmARF1-GFP recombinant construct and CaMV35S-GFP were transiently co-expressed in maize protoplast cells with a nuclear location signal (NLS) marker gene. The green fluorescence signal of ZmARF1-GFP exhibited colocalization with the nucleus, alongside the RFP fluorescence signal of the nuclear marker. Furthermore, it was also detected in the cytoplasm (Fig 2D). We further investigated the impact of low-phosphorus (Pi) stress on the nucleocytoplasmic translocalization of ZmARF1 in roots. It was observed that low-Pi stress influenced the nucleocytoplasmic abundance of ZmARF1 (Fig 2E). While low-Pi (LP) stress generally increased the abundance of ZmARF1 in both the cytoplasm and nucleus compared to high-Pi (HP) conditions, the accumulation of ZmARF1 protein in the nucleus was significantly higher under low Pi stress compared to the cytoplasm (Fig 2E). This observation suggests that LP stress may promote the nuclear localization of ZmARF1.

### 2.3 ZmARF1 is a positive regulator of low Pi stress response and root development in maize

**Fig 3:**
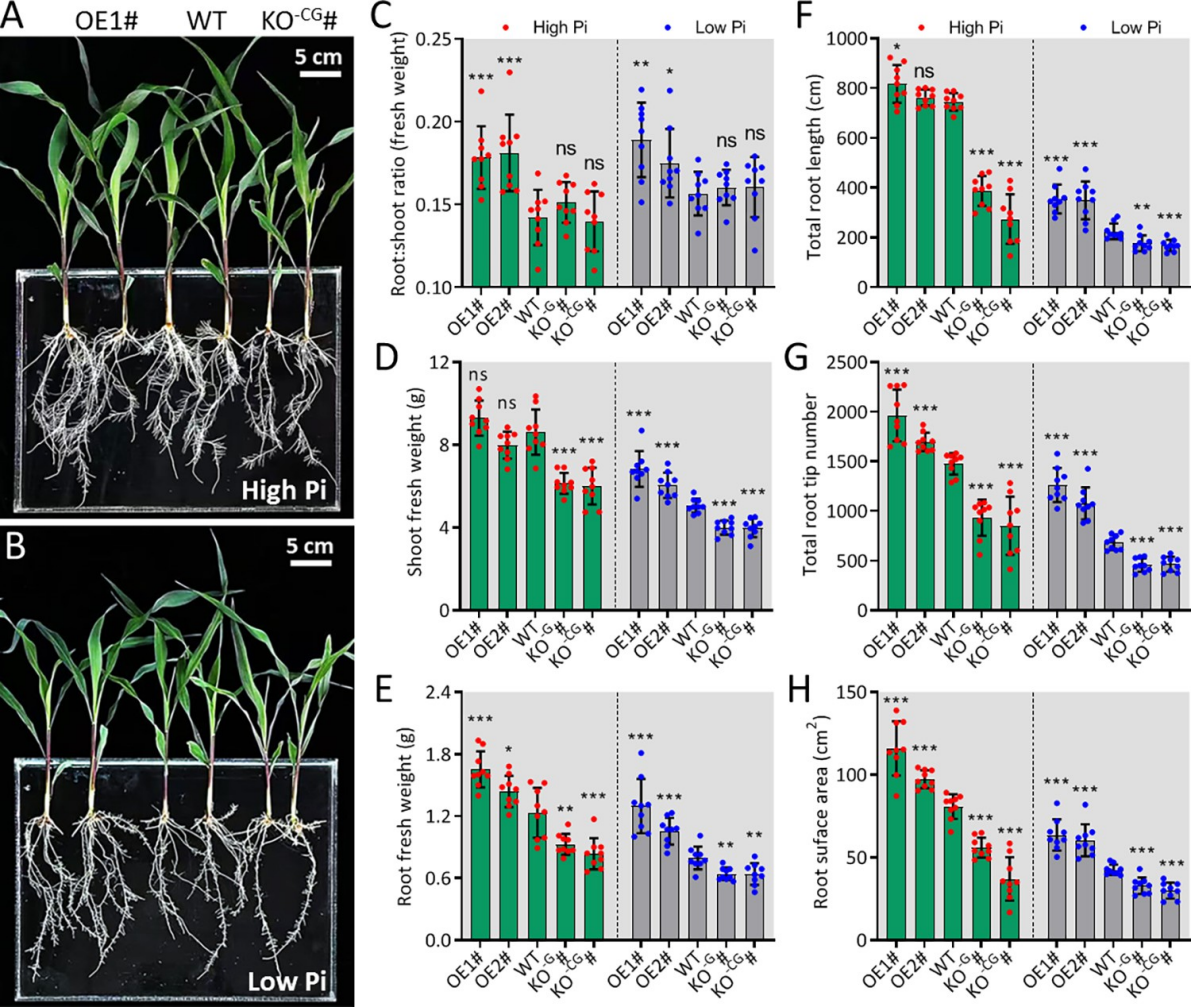
*ZmARF1* conferred Pi stress tolerance by promoting root development. Overexpression and knockout mutants of *ZmARF1*, and WT at the 3-leaf stage were transferred to (A) HP and (B) LP for 14 days and evaluated for growth and root development. Phenotypic traits: (C) Root-to-shoot ratio, (D) Shoot fresh weight, (E) Root fresh weight, (F) Total root length, (G) Total root tip number, and (H) Root surface area, were evaluated after HP and LP treatment. Three biological repeats were treated; more than 9 plants of each genotype were used for each biological repeat. Means are presented as bar graphs and error bars represent standard deviation. OE1# and OE2# represent the two overexpression lines, WT is the wild-type, KO^-G^# and KO^-CG^# are two knockout mutants. *, P < 0.05; **, P < 0.01; ***, P < 0.001; ns, not significant (Student’s *t* test).

To evaluate the biological function of *ZmARF1* in maize, we generated *ZmARF1* overexpression lines (S3 Fig) and their knockout mutants (S4 Fig) to test the phenotypic differences under various Pi supply levels. Two independent overexpression lines (OE1 and OE2), two *zmarf1* mutants (KO^-C^ and KO^-CG^), and wild-type (WT) at the 3-leaf stage were treated with HP or LP for 14 days. As anticipated, almost all traits among the different genotypes were affected by LP treatment, resulting in the inhibition of plant growth. The overexpression lines consistently showed improved growth and better root development under both HP and LP conditions compared to the WT. The root-to-shoot ratio and fresh shoot and root weights were enhanced in the overexpression lines compared to those in the WT, and these traits were impaired in the *zmarf1* knockout mutants (Fig 3C and 3D). Root phenotypic traits, such as root length, root tip, and root surface area, were higher in the overexpression lines than in the WT lines. In contrast, the knockout mutant *zmarf1* attenuated growth and root development (Fig 3), with a significant reduction in these root traits (Fig 3F–3J). These results suggested that *ZmARF1* positively regulates low-Pi stress tolerance in maize.

### 2.4 *ZmARF1* regulates Pi mobilization by modulating *PSI* gene expression

**Fig 4:**
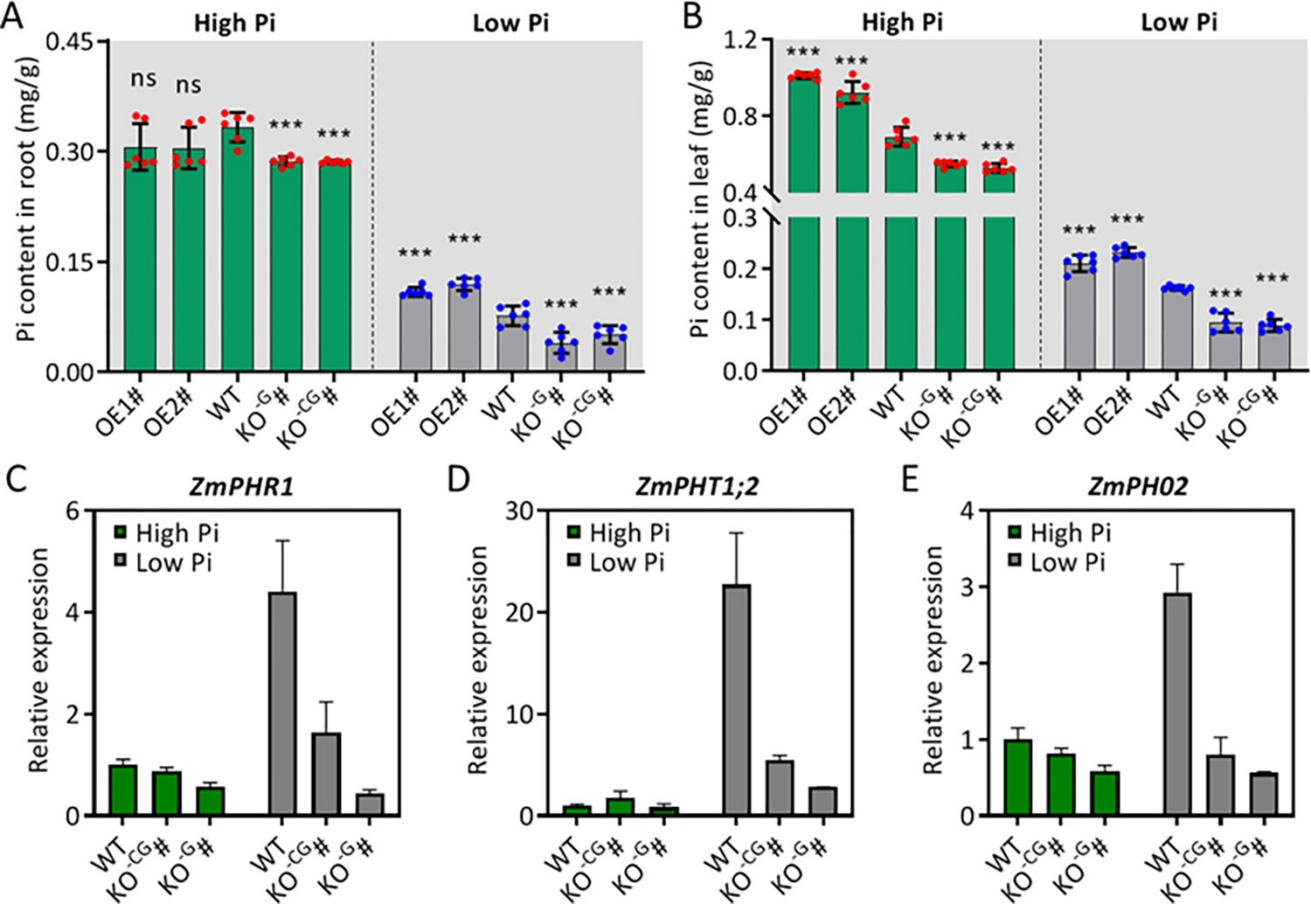
*ZmARF1* regulates Pi mobilization and PSI gene expression in maize. Pi content in (A) Root (B) Leaf of *ZmARF1* overexpression lines, *zmarf1* knockout mutants, and WT under HP and LP conditions. Three biological repeats were treated; more than nine plants of each genotype were used for each biological repeat. RT-qPCR analysis of (C) *ZmPHR1*, (D) *ZmPHT1;2*, and (E) *ZmPHO2* in knockout mutant *zmarf1* and WT under HP and LP conditions. Transcript levels of *ZmPHR1*, *ZmPHT1;2*, and *ZmPHO2* were measured relative to *ZmActin* and *ZmGADPH*. The experiment was repeated three times with similar results. Means are presented as bar graphs and error bars represent standard deviation. ***, P < 0.001; ns, not significant (Student’s *t* test).

Considering the phenotypic diversity of the different transgenic lines of *ZmARF1* under LP conditions, we speculated that *ZmARF1* might be involved in phosphorus absorption and translocation. Under HP conditions, there was no discernible difference in the root Pi content between the WT and two overexpression lines; however, these were higher than those in the mutant lines (Fig 4A). Under LP treatment, however, the root Pi concentration of the overexpression lines OE1# and OE2# was significantly higher than that of the WT, whereas the Pi concentration in the roots of the two knockout mutants, KO^-G^# and KO^-CG^#, was significantly lower than that of the WT (Fig 4A). In contrast, the two *ZmARF1* overexpression lines consistently recorded increased leaf Pi concentrations under both HP and LP conditions compared to the WT (Fig 4B). At the same time, the knockout mutants *zmarf1* had lower leaf Pi content than did the WT plants under both HP and LP conditions (Fig 4B). Plant adaptation to Pi-deficient environments is largely influenced by the activities of Pi transporter genes [33]. To ascertain the molecular mechanism underlying the role of *ZmARF1* in regulating low-Pi stress tolerance, RT-qPCR was used to analyze the transcript levels of conventional PSI genes, such as *ZmPHR1*, *ZmPHT1*;2, and *ZmPHO1;2* after HP and LP treatments. The results showed that The transcript levels of *ZmPHR1*, *ZmPHT1*;2, and *ZmPHO2* in the knockout mutant *zmarf1* were up to 5-folds lower than those in the WT after low-Pi treatment (Fig 4C–4E). These results imply that *ZmARF1* regulates the Pi stress-induced expression of *ZmPHR1*, *ZmPHT1*;2, and *ZmPHO2*, which are key regulators of the P-deficiency response and could, in part, account for the molecular mechanisms conferring Pi stress tolerance and maintaining Pi homeostasis. The regulation of *ZmPHR1*, *ZmPHT1*;2, and *ZmPHO2* significantly affects Pi mobilization in maize. These results suggest that *ZmARF1* plays a regulatory role in Pi homeostasis, likely by influencing the mobilization and translocation of phosphates from the source (roots) to the sink (shoots).

### 2.5 *ZmLBD1*—Is a downstream target of ZmARF1

**Fig 5:**
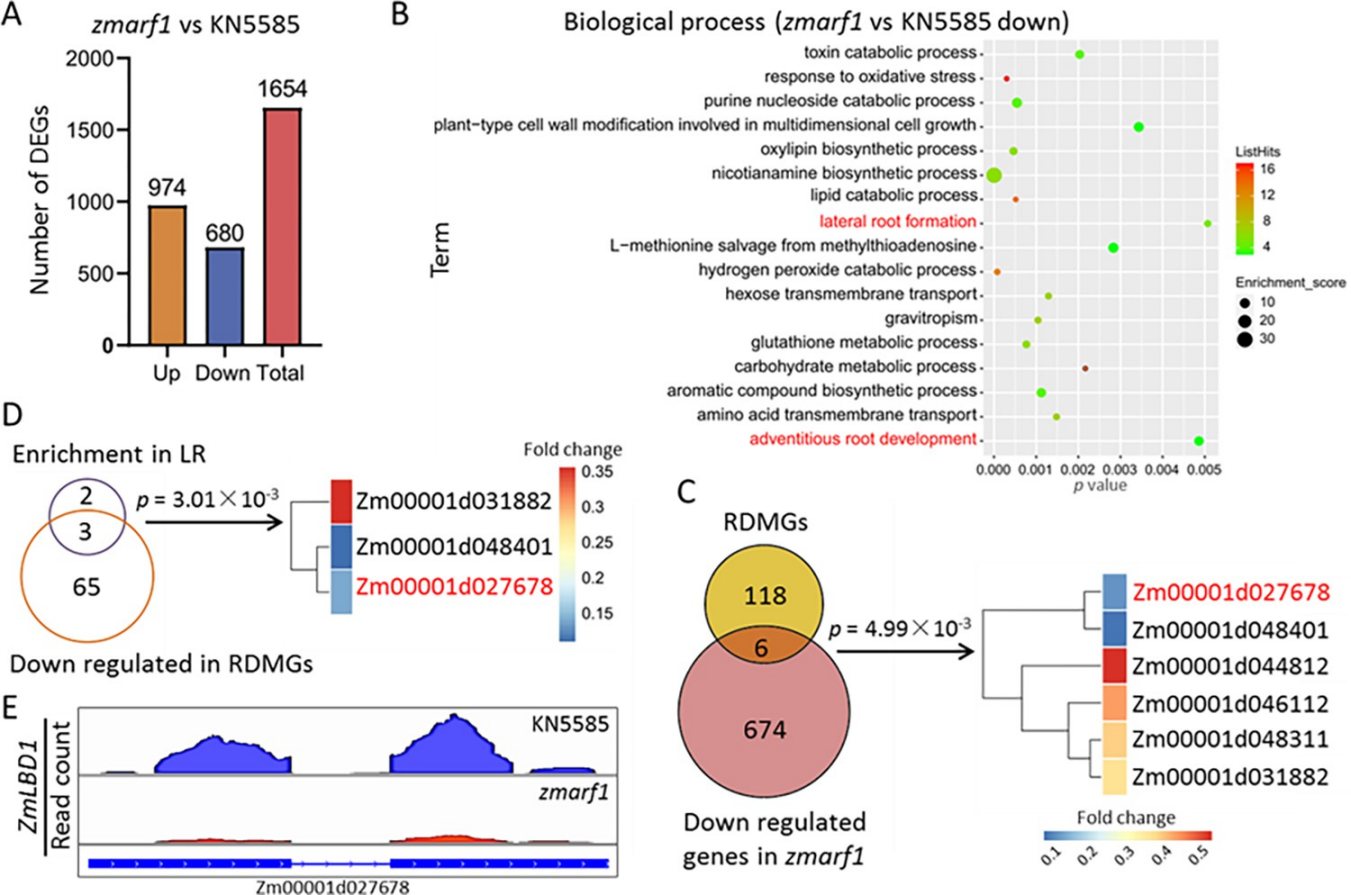
Transcriptome profiling revealed down regulated DEGs in knockout mutants of ZmARF1. (A) Differentially expressed genes between zmarf1 knockout mutant and KN5585 (WT). (B) Functional assignment of the DEGs by GO analysis, bar represents q value. (C) Overlap of down regulated DEGs in zmarf1 with genes involved in lateral root formation in maize. Fisher’s exact test was used to calculate the p value. Scale represents fold change. (D) Venn diagram showing overlapped down regulated DEGs in zmarf1 with RDMGs in maize. Fisher’s exact test was used to calculate the p value. (E) Visualization of RNA-seq coverage profile for ZmLBD1 revealed by the Integrated Genome Viewer browser. Scale represents normalized read counts.

To unravel the molecular mechanisms governing the role of *ZmARF1* in regulating root growth and development, RNA-seq analysis was performed using the root tissues of KN5585 (WT) and *zmarf1* knockout mutants. A total of 1654 differentially expressed genes (DEGs) were identified between KN5585 and *zmarf1* with a 2-fold cutoff (false discovery rate <0.05) (Fig 5A). Among these, 680 (41%) were downregulated and the remaining 974 (59%) were upregulated (Fig 5A). Gene Ontology (GO) enrichment analysis revealed a wide array of signaling-mediated biological processes, including responses to oxidative stress and plant-type cell wall modifications involved in multidimensional cell growth, lateral root formation, gravitropism, and adventitious root development (Figs 5B and S5). The collection of root development marker genes (RDMGs) in maize comprised 124 genes involved in various regulatory networks underlying root development [34]. Furthermore, 8% (10/124) of the RDMGs were present among the DEGs in the *zmarf1* mutant (S6A Fig). In line with the transcript of *ZmARF1* in the *zmarf1* mutant (S4C Fig), six and four of the ten differentially expressed RDMGs were downregulated and upregulated, respectively (Figs 5C and S6B–S6F). The transcripts of the six down-regulated DEGs were validated using RT-qPCR (S7 Fig). Three of the downregulated RDMGs, *ZmLBD1* (Zm00001d027678), *ZmRTCL1* (Zm00001d048401), and LOB domain-containing protein 29 (Zm00001d031882), were enriched in lateral root (LR) formation (Fig 5C). The LBD family of proteins is implicated in the regulation of lateral root development in plants [30]. Additionally, the integrative genomics viewer (IGV) with the RNA-seq data showed that, compared with the WT, the transcript levels of *ZmLBD1* were significantly repressed in *zmarf1* (Fig 5E), in line with the transcript of *ZmARF1* in the *zmarf1* mutant (S4C Fig). Transcriptomic profiling suggests that *ZmLBD1* is a potential downstream target of *ZmARF1*.

### 2.6 ZmARF1 binds to the GC-box motif in the promoter of *ZmLBD1* and directly activates its transcription *in vivo*

**Fig 6:**
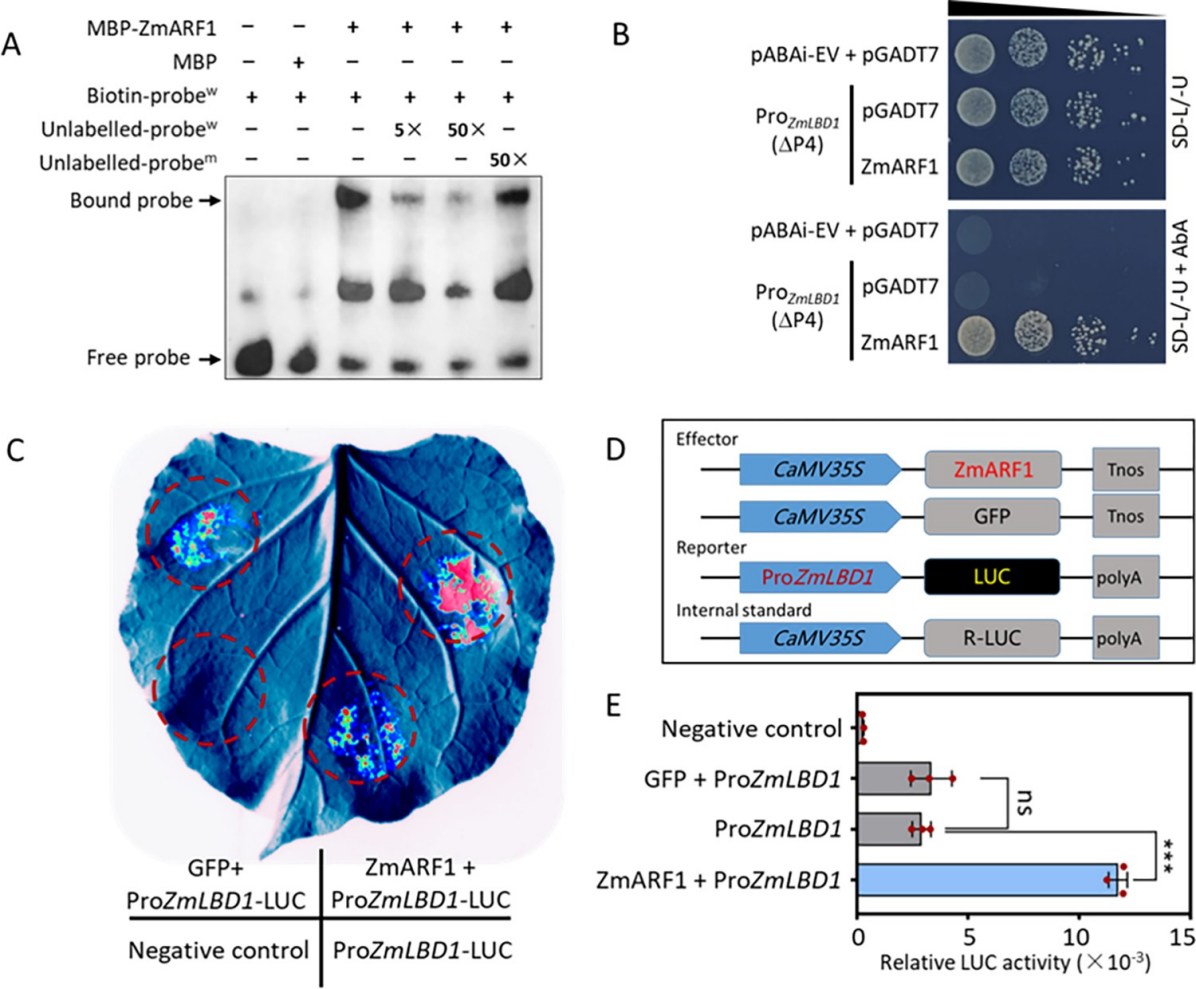
*ZmARF1* binds to the promoter of *ZmLBD1* and activates its transcription. (A) EMSA reveals specific binding of *ZmARF1* to the GC-box motif in the promoter of *ZmLBD1*. Superscript w and m indicate wild-type and mutant GC-box competitors, respectively. (B) Y1H yeast strain was transformed with pABAi-Pro:ZmLBD1 construct and selected on either SD/-L/-U or SD/-L/-U + 200 ng ml^-1^ AbA. The empty vector pGADT7 serves as a control for the effect factor ZmARF1. (C) Transient co-expression of ZmARF1-GFP or GFP effectors with the Pro:ZmLBD1:LUC reporter in *N*. *benthamiana* leaves. (D) Schematic representation of the effector and reporter constructs used for transient expression in *N*. *benthamiana* leaves. (E) The LUC/REN ratio representing the relative activity of *ZmLBD1* promoter. The experiment was repeated three times with similar results. Each column represents the mean of three independent samples, and error bars represent standard deviation. Significant differences between means were determined using students *t*-test. ***, P<0.001; ns = no significant difference.

The DNA-binding domain (DBD) of ARFs allows them to recognize and interact with a conserved auxin response element (AuxRE) in the promoters of target genes to regulate transcription [35]. An electrophoretic mobility shift assay (EMSA) was performed to ascertain whether *ZmARF1* has a DNA-binding affinity to *ZmLBD1* promoter. Although PLANTCARE analysis of the ~2.5 kb promoter region of *ZmLBD1* did not identify any AuxREs, numerous GC box motifs were identified and selected. Electrophoretic mobility shift assay was conducted using an MBP-ZmARF1 recombinant protein and a biotin-labeled GC box motif. The results showed that recombinant MBP-ZmARF1 could bind to the biotin-labeled GC box probe, as revealed by the presence of a supershifted band when recombinant MBP-ZmARF1 was incubated with the labeled probe (Fig 6A). In contrast, no supershifted bands were detected in the control reaction comprising only the labeled probe or the labeled probe incubated with MBP. A decrease in the supershift band was observed with an increase in the concentration of the unlabeled GC-box motif competitor (5× > 50×) (Fig 6A). In contrast, the addition of the mutant GC-box motif as a competitor to the labeled probe did not show any discernible effect on the shifted band (Fig 6A), suggesting that *ZmARF1* binds specifically to the GC-box motif in the promoter of *ZmLBD1*.

We performed a yeast one-hybrid (Y1H) assay to determine whether *ZmARF1* exhibited transcriptional activation. The full-length coding sequence (CDS) of *ZmARF1* was cloned into the pGADT7 vector to generate the prey construct. Because of the powerful auto-activity of the 2.5 kb promoter region of *ZmLBD1* upstream of the start codon (ATG), it was not suitable to directly perform the Y1H assay as a bait. The 2.5 kb promoter region of *ZmLBD1* was divided into five fragments of approximately 500 bp each (S8A Fig). Two of the five highly active promoter fragments were not inhibited by AbA (S8B Fig). The fourth fragment (ΔP4) contained a GC-box, whose self-activity was inhibited by AbA, was used for verification in Y1H. The results showed that yeast cells harboring pABAi-ProZmLBD1 and the effect factor *ZmARF1* grew normally on SD/-Leu/-Ura and SD/-Leu/-Ura + 200 ng ml^-1^ AbA, whereas those transformed with the empty pGADT7 plasmid grew on SD/ -Leu/-Ura but not on SD/-Leu/-Ura + 200 ng ml^-1^ (Fig 6B). The results of the Y1H assay confirmed that *ZmARF1* directly interacts with the promoter of *ZmLBD1* and transcriptionally regulates expression of *ZmLBD1*. To confirm that *ZmARF1* modulate the transcription of *ZmLBD1*, we performed a dual-luciferase transcription activation assay using *Nicotiana benthamiana* leaves. ZmARF1-GFP or GFP were transiently co-expressed with the *ProZmLBD1*:LUC reporter in *N*. *benthamiana* leaves (Fig 6C). The expression of Renilla (REN) was used as an internal control (Fig 6D). Coexpression of *ProZmLBD1*:LUC and *ZmARF1* effectors significantly increased luciferase activity compared to coexpression with the GFP effector or expression of the ProZmLBD1:LUC reporter alone (Fig 6C and 6E). This suggested that *ZmARF1* acts as a positive regulator of *ZmLBD1* and possesses transcriptional activation activity.

### 2.7 Target gene *ZmLBD1* regulates root development

**Fig 7:**
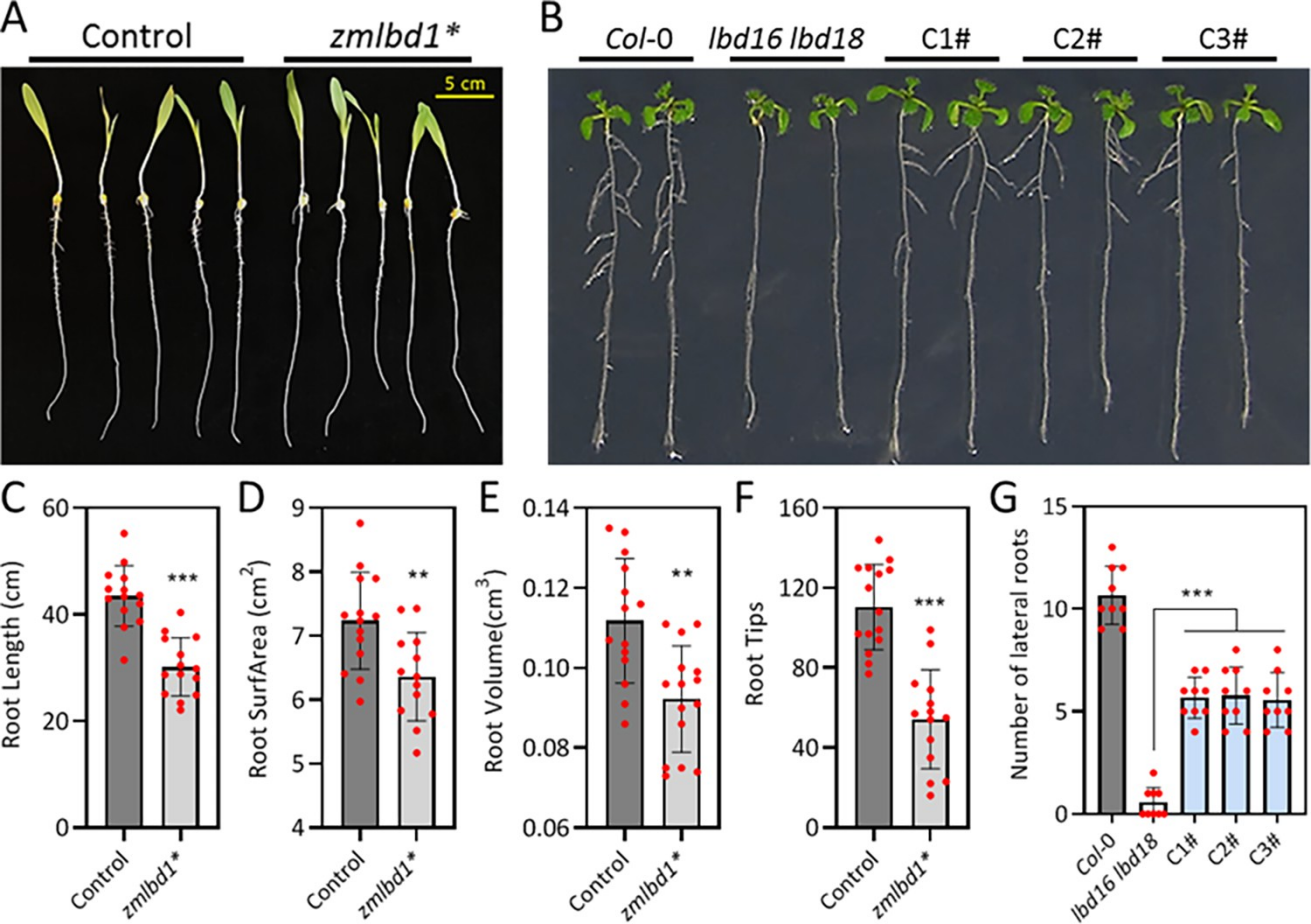
ZmLBD1 regulates root phenotypic traits. (A) Root phenotypic traits of WT and zmlbd1 mutant of maize at seedling stage. (B) Overexpression of ZmLBD1 partially restored lateral root growth in lbd16 lbd18. C1#, C2# and C3# represent the three overexpression lines of ZmLBD1 in lbd16 lbd18 mutant. (C) Root length, (D) Root surface area, (E) Root volume, and (F) Root tips were compared between the WT and zmlbd1 mutant. Each was replicated three times, using more than ten plants of each genotype for each replication. (G) The statistical analysis of lateral roots number is from (B). The bar graphs show the means and error bars represent standard deviation. Significant differences between means were determined using Student’s t-test. **, P < 0.01; ***, P < 0.001; ns = not significantly different. Three biological repeats were carried out; more than 9 plants of each genotype were used for each biological repeat.

To characterize the biological functions of *ZmLBD1* in maize, an EMS induced point mutant of *ZmLBD1* was generated. Genotyping showed that the point mutation in the exon of *ZmLBD1* was a nonsense mutation that formed a terminator (S9A Fig), resulting in altered root morphological traits in *zmlbd1* maize mutant (Fig 7A). Root morphology-related traits in the *zmlbd1* mutants, such as root length, root surface area, root volume, and root tip number, were significantly reduced compared to those in the WT (Fig 7C–7F). Moreover, overexpression of *ZmLBD1* in *lbd16lbd18* double mutants exhibited a significant reduction in lateral roots and partially restored lateral root growth in Arabidopsis **(**Fig 7B and 7G). Genotyping of all the tested lines is shown in S9B Fig. These results confirm that *ZmLBD1* plays a significant biological role in regulating root growth and development. The role of *ZmLBD1* in root development aligns with that of *ZmARF1* in the present study. This suggests that *ZmARF1* regulates root development through its interaction with and transcriptional regulation of *ZmLBD1*.

### 2.8 ZmERF2 binds to the ABRE motif in promoter of *ZmARF1* and represses its transcription

**Fig 8:**
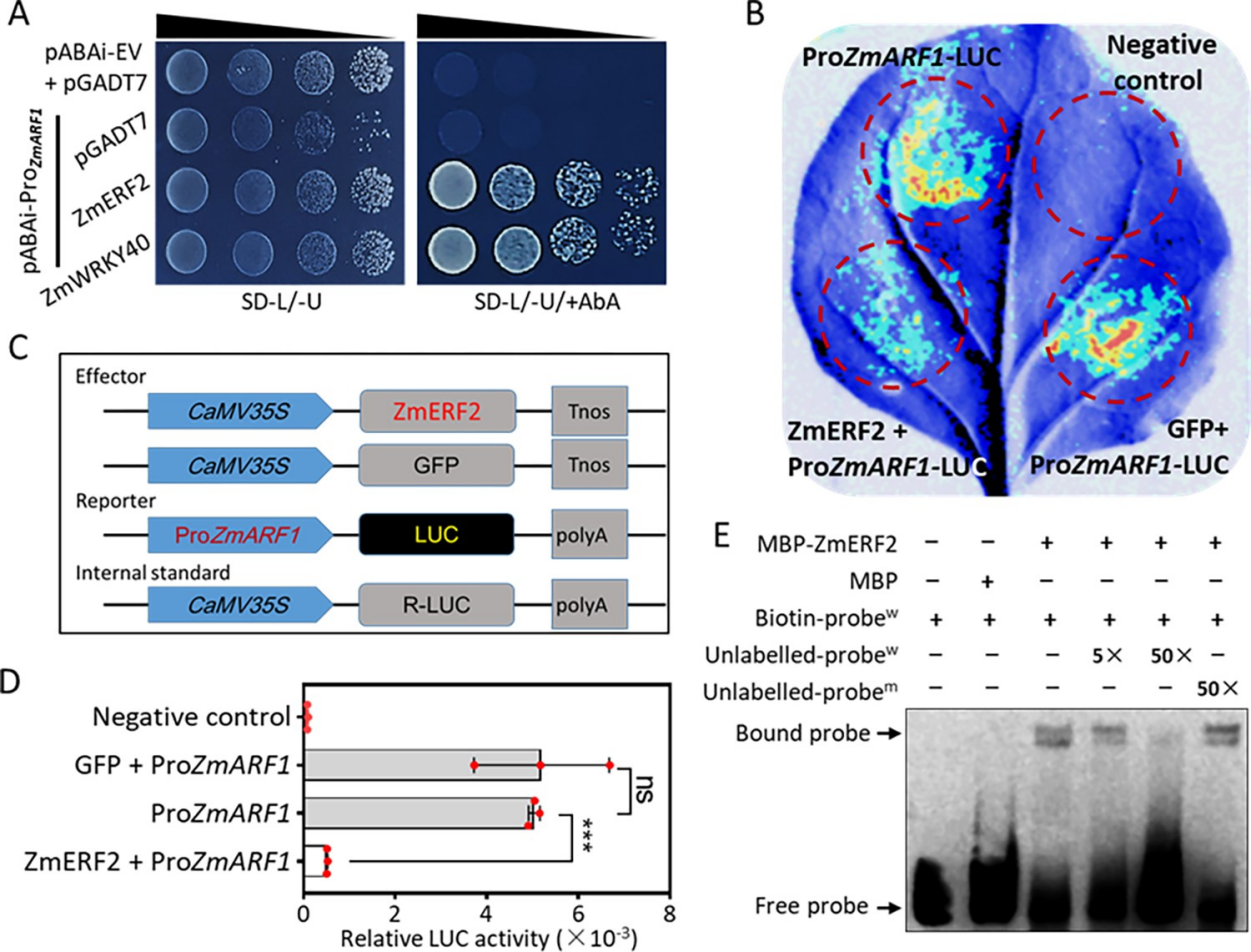
*ZmERF2* interacts with promoter of *ZmARF1*. (A) Y1H assay confirms *ZmERF2* and *ZmWRKY40* interact with promoter of *ZmARF1* in yeast. (B) Transient co-expression of ZmERF2-GFP or GFP effectors with the ProZmARF1:LUC reporter in *N*. *benthamiana* leaves (C) Schematic representation of the effector and reporter constructs used for dual luciferase assay (D) The LUC/REN ratio representing the relative activity of *ZmARF1* promoter. Each column represents the mean of three independent samples, and error bars represent standard deviation. Significant differences between means were determined using students *t*-test. ***, P<0.001; ns = not significantly different. The experiment was repeated three times with similar results. (E) EMSA reveals that *ZmERF2* binds specifically to ABRE motif in promoter of *ZmARF1*.

To enhance our understanding of the molecular mechanisms governing the biological function of *ZmARF1*, Y1H library screening was performed to identify transcription factors that target the promoter of *ZmARF1*. A 2kb promoter sequence of *ZmARF1* was cloned into pABAi to obtain the bait construct pABAi-ProZmARF1. The pABAi-ProZmARF1 bait construct was used to transform Y1H Gold-competent yeast cells and tested for AbA resistance (S10A Fig). Yeast cells harboring the pABAi-ProZmARF1 bait construct were transformed with the pGADT7 (AD) prey fused with a cDNA library plasmid and screened on SD/-U + 300 ng mL^-1^ AbA and resulting single colonies were subjected to PCR amplification and verified by gel electrophoresis (S10B Fig). Candidate genes annotated as transcription factors (ZmERF2 and ZmWRKY40) were selected as potential interacting proteins (S4 Table). To confirm the binding interaction between the selected candidate genes and the promoter of *ZmARF1*, the full coding sequences of *ZmERF2* and *ZmWRKY40* were cloned into the pGADT7 vector. Yeast cells carrying ProZmARF1 grew normally on SD/-L/-U +300 ng mL^-1^ of AbA when transformed with either pGADT7-ZmERF2 or pGADT7-ZmWRKY40 but not with pGADT7 (Fig 8A). This demonstrates that ZmERF2 and ZmWRKY40 interact with the promoter of *ZmARF1*.

The DNA-binding activity of ZmERF2 to the promoter of *ZmARF1* was confirmed using EMSA. Analysis of the 2 kb promoter region of *ZmARF1* identified among other *cis*-acting elements, the ABRE motif. EMSA was performed using MBP-ZmERF2 recombinant protein, biotin-labeled ABRE motif, unlabeled ABRE, or mutated ABRE competitors. *ZmERF2* bound normally to the ABRE-labeled probes, as revealed by the supershifted band (Fig 8E). In contrast, no supershifted bands were detected in the control reaction comprising only the labeled probe or the labeled probe incubated with MBP (Fig 8E). There was a decrease in the supershifted band with increasing concentrations of the unlabeled ABRE motif competitor (Fig 8E). In contrast, the mutant ABRE motif did not affect the supershift when added as a competitor to the biotin-labeled ABRE motif probe (Fig 8E), suggesting that *ZmERF2* specifically binds to the ABRE motif in the promoter of *ZmARF1*. The transactivation of *ZmERF2* was evaluated using a dual-luciferase assay. The ZmERF2-GFP or GFP effectors were transiently co-expressed with the ProZmARF1:LUC reporter in *N*. *benthamiana* leaves (Fig 8B). The expression of Renilla (REN) was used as an internal control (Fig 8C). Co-expression of Pro*ZmARF1*:LUC and ZmERF2-GFP effectors significantly reduced luciferase activity compared to co-expression with the GFP effector or expression of the ProZmARF1:LUC reporter alone (Fig 8B and 8D). This suggests that *ZmERF2* acts as a negative regulator and represses the transcription of *ZmARF1*.

## 3. Discussion

### 3.1 Polymorphic sites in *ZmARF1* is associated to Pi stress tolerance traits in maize

Candidate gene-based association analysis has been very useful for testing the association between SNPs within target genes and phenotypic traits [36,37], and allows for the identification of one or multiple alleles in target genes that create variations in traits of interest. In the current study, candidate gene-based association analysis was used to identify significant associations between SNP and haplotype polymorphisms of the entire length of the *ZmARF1*, and phosphorus deficiency tolerance traits in maize. A total of 81 SNPs were identified in the 15432 bp full-length sequence of *ZmARF1* (S2 Table). The average SNP frequency among the 81 detected SNPs was one SNP per 191 bp (S2 Table). The SNP frequency detected in the present study was within the range reported for plants in the Poaceae family, indicating a high level of genetic diversity in the *ZmARF1* sequence, which is associated with phosphorus deficiency tolerance traits in maize. For example, one SNP per 60.8 bp was observed in elite maize inbred lines [38], and one SNP per 200 bp was detected in 977 unigenes in barley, based on genes responsive to abiotic stress [39]. The presence of such SNP could permit an LD-based approach for trait dissection and gene mapping in maize [40].

Moreover, 29 of the 81 identified SNPs in the introns were completely linked with SNPs in the exons and formed a large linkage disequilibrium (LD) block (Fig 1B). LD is a measure of nonrandom associations between alleles at different loci within the genome. The presence of LD is a prerequisite for association mapping of genes related to phenotypes [41,42]. Association studies centered on LD offer the possibility of high-resolution identification of genes that contribute to phenotypic variation [43,44]. The phosphorus tolerance traits investigated in this study are crucial for determining the tolerance of maize to phosphate stress. Therefore, the presence of LD blocks in *ZmARF1* makes it possible to identify the nucleotide diversity in *ZmARF1* associated with low-P tolerance traits using a general linear model (GLM), a GLM fitted with population structure (GLM + Q), and a mixed linear model (MLM, fused with Q+K). Polymorphisms in *ZmARF1* were significantly associated with plant height, TRT number, and total root length under LP conditions (Figs 1, S1 and S2). Association mapping between polymorphic sites in genes and low-phosphorus tolerance traits has been established [45–47]. Therefore, association mapping is a reliable tool to identify specific genes or gene regions that underlie specific plant traits or responses in plants [48–50]. The results of *ZmARF1* based association mapping in this study established an association between the polymorphic sites of *ZmARF1* and some low Pi stress tolerance traits, suggesting that *ZmARF1* could regulate low phosphorus stress responses in maize.

### 3.2 *ZmARF1* confers Pi stress tolerance and regulates root development in maize

Plants increase Pi acquisition under Pi stress by modifying their root morphological traits, including shallow primary roots and increased adventitious and lateral roots, under Pi stress [2,51]. In the present study, analysis of Pi-deficient phenotypes revealed that overexpression of *ZmARF1* significantly improved tolerance to Pi stress, as evidenced by better growth performance and root development compared to the WT, in sharp contrast to the phenotype of the knockout mutants (Fig 3). Previous studies have revealed that increased root traits, such as root length, root surface area, and root tip number, increased Pi uptake and conferred Pi stress tolerance owing to the increased area between the roots and soil, which optimized Pi mobilization under P-deficient conditions [52,53]. Due to the immobility of phosphorus, the development of fine roots and increased root surface area increase Pi absorption in P-deficient environments [54]. Active participation of genes in the Pi stress response through root remodeling has been reported [55,56]. Genes encoding transcription factors belonging to the WRKY, MYB, ZFP, and ARF families, and their non-coding RNAs actively participate in Pi stress regulation in plants [57]. The roles of some ARF proteins in the regulation of phosphorus stress have also been reported [27,28,58]. This further confirmed the role of *ZmARF1* as a positive Pi stress response transcription factor in maize.

*ZmARF1* targeted *ZmLBD1* and it positively regulated its transcription (Fig 6A–6E) and thus promoted root development (Fig 7). *ZmARF1* has a DNA-binding domain at its N-terminus through which it interacts with its downstream targets in order to regulate transcription. The transcript levels of *ZmARF1* and *ZmLBD1* were both significantly downregulated in the *zmarf1* knockout mutants (Figs 5C–5E and S4C), suggesting that the biological function of *ZmARF1* in root development (Fig 3) could be aligned with the function of *ZmLBD1* (Fig 7). Root phenotypic traits were significantly reduced in the knockout mutants of *zmarf1* and *zmlbd1*, suggesting that the transcription regulation of *ZmLBD1* by *ZmARF1* contributes significantly to the processes that lead to root development in maize. A nexus exists between the root development and Pi acquisition in plants [52], hence the role of *ZmARF1* in root development is to promote Pi acquisition in maize. Ethylene biosynthesis is a transcriptional and post-transcriptional regulated process [59] that modulates stress responses in plants [60]. The ethylene response pathway has been reported to negatively regulate low-Pi stress responses and root development in plants [61,62]. This reported information is also consistent with our finding that *ZmERF2* negatively regulated the transcription of *ZmARF1* (Fig 8A–8E).

**Fig 9:**
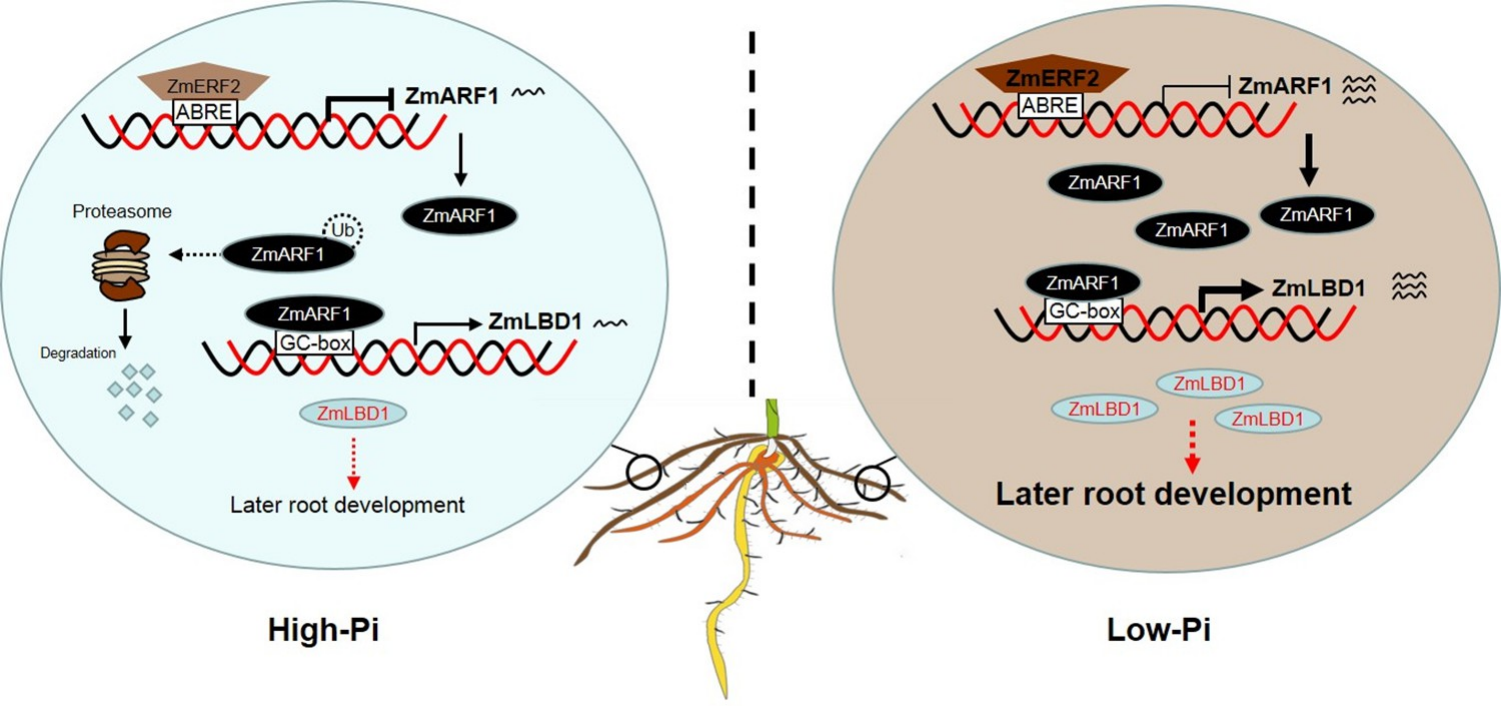
A working model for the *ZmERF2*-*ZmARF1*-*ZmLBD1* signaling module in response to low-Pi stress. The graph on left shows that maize has less lateral root growth under high phosphorus conditions. *ZmARF1* is inhibited from regulating *ZmLBD1* and lateral root development by *ZmERF2* by ubiquitination and degradation. On the contrary, the graph on the right shows the promotion of lateral root development in maize under low-phosphorus conditions. Transcriptional repression of *ZmARF1* by *ZmERF2* is limited under low Pi, abundance of ZmARF1 results in an increased *ZmLBD1* transcription and consequently lateral root development.

Based on the findings of the present study, we have proposed a *ZmERF2*-*ZmARF1*-*ZmLBD1* regulatory network that coordinates the nexus between root development and low-Pi stress response in maize (Fig 9). Under high Pi conditions, there may be a balance between post-translational degradation and the transcription of *ZmARF1*, which is mediated by *ZmERF2*, leading to the repression of *ZmLBD1* transcription and a reduction in lateral root development. Under low-Pi conditions, increased *ZmARF1* expression resulted in increased *ZmLBD1* transcription, resulting in increased lateral root development to adapt to the physiological environment of phosphorus deficiency.

### 3.3 ZmARF1 is a positive regulator of *PSI* genes and Pi homeostasis

The molecular mechanism governing the role of *ZmARF1* in phosphate stress tolerance was further investigated through the differential expression of PSI genes using RT-qPCR (Fig 4C–4E). Phosphorus mobilization from soil is primarily a function of plant roots and directly involves Pi transporters that are precisely controlled at the transcript and protein levels [63]. In the present study, RT-qPCR revealed alterations in the expression of Pi stress-inducible genes *ZmPHR1*, *ZmPHT1;2* and *ZmPHO*2. Pi-inducible genes belonging to the phosphate transporter (PHT) [64], phospahte1 (PHO1) [65], and phosphate starvation response (PHR) [66] families are upregulated during Pi deficiency and are considered useful marker genes in the Pi signaling pathway. Some high-affinity Pi (PHT1) family transporters play a major role in Pi uptake from the soil to roots, whereas some PHO1 family proteins increase root Pi xylem loading [19]. OsPHR2 regulates root hair proliferation and elongation, suggesting that OsPHR2 is involved in both systematic and local Pi signaling pathways [67]. Only a few ARFs have been implicated in the regulation of Pi stress, most of which involve the regulation of Pi stress-inducible genes. For example, overexpression of *ZmARF4* enhances lateral root growth, Pi mobilization, and expression of the responsive gene *AtANS1* [27]. Knockout of *OsARF16* reduces *PSI* gene expression [68]. Similarly, in the present study, there was a decrease in Pi content in the roots and leaves of knockout mutants under LP conditions, whereas overexpression of *ZmARF1* significantly increased these traits (Fig 4A and 4B). Knockout of *ZmARF1* drastically reduced the expression of *ZmPHR1*, *ZmPHT1;2* and *ZmPHO*2 in *zmarf1* mutant maize lines relative to the WT under low-Pi conditions (Fig 4C–4E). This result is in tandem with previous findings; for example, Pi content and uptake were increased by the overexpression of *PHT1;1* and decreased in *pht1;1* mutant [69]. An increase in the transcript levels of the MYB-related transcription factor PHR1 in *phr1* mutants and in WT Arabidopsis overexpressing *PHR1* significantly increased Pi uptake [70]. *PHO1* regulation by WRKY6 is Pi-dependent, and low Pi treatment may inhibit *PHO1* expression by WRKY6 in Arabidopsis [71]. This suggests that the increase in Pi content in the transgenic lines overexpressing *ZmARF1* is strongly linked to the upregulation of *ZmPHT1;2*, *ZmPHR1* and *ZmPHO2* in the transgenic lines. These results suggested that *ZmARF1* regulates Pi homeostasis under LP conditions by regulating the expression of Pi stress-inducible genes.

## 4. Materials and methods

### 4.1 Plant transformation in maize

The full-length coding sequence (CDS) of *ZmARF1* was amplified by PCR from the low Pi-tolerant P178 maize inbred line and it was cloned into the *Bam*H I and *Sac*I sites of the CUB expression vector driven by the maize ubiquitin promoter with a 3×Flag tag at the N-terminus. The gene-specific primers used in this study are stated in S1 Table. The fusion construct was confirmed by sequencing and sent to WIMI Biotechnology (Jiangsu, China) for transformation into the maize KN5585 background. Positive transformants harboring the Ubi::ZmARF1-Flag construct were screened and selected using Basta (Coolaber, Beijing, China) until homozygous overexpression (OE) lines were obtained from the T_4_ generation. Homozygous lines with relatively high expression of *ZmARF1* were detected by western blotting using anti-Flag anti body (S3 Fig). Transgenic lines with relatively high expression of *ZmARF1* were selected for subsequent experiments.

The CRISPR-GE web tool was used to design an sgRNA sequence with the highest efficiency and lowest number of off-targets to target the 10^th^ exon located on the AUX/IAA functional domain in the open reading frame (ORF) of *ZmARF1* (S4A Fig). We amplified the sgRNA by PCR using sequence-specific primers (S1 Table) and cloned it into the Cas9 vector at two *Hin*dIII sites. The construct plasmid of Cas9-*sgRNA* was sent to WIMI Biotechnology (Jiangsu, China) for transformation into a maize KN5585 background. Two homozygous mutant lines with different genome edits were selected (S4B Fig) for subsequent experiments.

The M_3_-generation seeds (EMS4-003556) of the EMS induced *zmlbd1* maize mutant were obtained from Elabcaas (https://elabcaas.cn/memd/public/index.html#/) [72]. DNA was extracted from leaves and subjected to PCR using a gene-specific primer (S1 Table). PCR products were sequenced and aligned to the B73 reference sequence.

### 4.2 Generating complementary lines of *ZmLBD1* in Arabidopsis double mutant *lbd16lbd18*

*ZmLBD1* is overexpressed in *lbd16 lbd18* double mutants [73]. The full-length CDS of *ZmLBD1* was amplified by PCR and cloned into the *Bam*H I and *Xba*I sites of the PCAMBIA2300-GFP expression vector. The primers used are indicated in S1 Table. Successful transformations were confirmed by G418 antibiotic screening and PCR amplification using the primers specified in S1 Table. Homozygous lines in the T_4_ generation that exhibited the highest expression of *ZmLBD1* were selected for phenotypic evaluation.

### 4.3 Plant materials and stress treatment

The maize (*Zea mays* L.) inbred line KN5585 was used as the WT for physiological experiments and all genetic transformations involving *ZmARF1*. Low Pi-tolerant (P178) and low Pi-sensitive (9782) inbred lines were used for expression analysis of *ZmARF1* after low Pi treatment. During the post-germination stage, uniform seedlings of the KN5585 (WT), *ZmARF1*-OE, and *zmarf1* knockout mutants were grown in a greenhouse for 14 days at 28°C and a 16/8 h light/ dark cycle in a modified Hoagland solution. Normal nutrient solution with high-Pi (HP) contained 2 M KNO_3,_ 2 M Ca(NO_3_)_2_.4H_2_O, 2 M MgSO_4_.7H_2_O, 1 M NH_4_H_2_PO_4,_ Na.FeEDTA, H_3_BO_3,_ MnCl_2_.4H_2_O, ZnSO_4_.7H_2_O, CuSO_4_.5H_2_O, and H_2_MoO_4_.H_2_O. For the low-Pi (LP) nutrient solution, 10 mM NH_4_H_2_PO_4_ was used. Seedlings were evaluated for growth, root phenotypic traits, differential gene expression, and Pi content.

### 4.4 Association analysis of polymorphisms on *ZmARF1* with low-Pi tolerant traits in maize

In previous studies, two association panels comprising 368 and 513 inbred maize lines were genotyped via RNA sequencing and the MaizeSNP50 BeadChip containing 56,110 SNPs (Illumina, San Diego, CA) [74,75], respectively. Polymorphic SNPs were extracted based on the physical localization of *ZmARF1* in the maize chromosomal genome. Informative SNPs with minor allele frequency ≥0.05 and missing rate of <25% were calculated for further analysis. In the present study, 356 inbred maize lines were evaluated for low-phosphorus stress tolerance at the seedling stage. Associations between the 13 tested root traits at the seedling stage under high- and low-Pi conditions [45–47] and polymorphic sites in the *ZmARF1* sequence were performed using TASSEL version 5.0, using the general linear model (GLM), GLM corrected using controlling population structure (GLM + Q), and a mixed linear model (MLM, fused with Q+K) [76]. The population structure (Q) and kinship (K) matrices were estimated as previously described [47].

### 4.5 Subcellular localization of *ZmARF1* in maize protoplast

The full-length CDS of *ZmARF1*, without a stop codon was amplified by PCR using gene-specific primers (S1 Table). The resulting PCR product was purified and fused to the *Sac*I and *Bam*HI sites in the HBT95-eGFP expression vector. Protoplasts were isolated from the leaf sheaths of etiolated leaves at the 3-leaf stage following the procedure described by Cao and Yao [77], with a few slight modifications. Plasmids HBT95-ZmARF1-GFP and HBT95-GFP were transformed into maize protoplasts together with nuclear localization signals (NLSs) fused with RFP, using polyethylene glycol (PEG) mediated transformation [78] with slight modifications. The resulting GFP and RFP fluorescence of the fusion constructs and NLS was detected using a laser confocal microscope (LSM800, ZEISS, Germany).

### 4.6 Measurement of root phenotypic traits

Root images were captured using the WinRhizo Pro 2008a image analysis system (Regent Instruments Inc., Quebec, Canada) after the respective treatments and growth conditions. Root phenotypic traits, including root length, surface area, volume, and tip length, were computed using the WinRhizo Pro 2008a image analysis system.

### 4.7 RNA extraction and RT-qPCR

The total RNA was extracted from leaf and root tissues after HP and LP treatments using TRIzol reagent (Invitrogen, Carlsbad, CA, USA) according to the manufacturer’s instructions. RNA (~1 μg) was used to synthesize first-strand cDNA using a reverse transcription kit (Vazyme, Nanjing, China) according to the manufacturer’s instructions. cDNA was diluted five-fold with nuclease-free water. RT-qPCR was performed using TransScript II Green One-Step qRT-PCR SuperMix (TransGen, Beijing, China), according to the manufacturer’s instructions. The housekeeping genes *ZmActin2* and *ZmGAPDH* were used as internal controls. The primers used are listed in S1 Table.

### 4.8 Protein extraction and immunoblot analysis

Leaf tissues were sampled from the WT and *ZmARF1*-OE lines at T_4_ for immunoblotting. Proteins were extracted using a cell lysis buffer comprising 50 mM TRIS pH 7.5, 150 mM NaCl, 0.2% NP40, 0.1% Triton X-100, and 1 mM PMSF. Plant samples were ground into a fine powder in liquid nitrogen and approximately 0.05 g was homogenized in 100 μL of cell lysis buffer. The separation of nuclear and cytoplasmic proteins from both WT and ZmARF1-overexpressed roots was conducted using the same method as previous described [79]. The protein extract (10 μL) was separated by electrophoresis on a 10% SDS-polyacrylamide gel and transferred onto a polyvinylidene difluoride membrane (Bio-Rad, 1620256#). Membranes were immunoblotted with an anti-Flag antibody (F3165 #; Sigma-Aldrich). The nuclear and cytoplasmic proteins were detected with anti-Histone (H3) and anti-Actin as internal control, respectively. Proteins were visualized using an enhanced chemiluminescence kit (Thermo Fisher Scientific, 32209).

### 4.9 Measurement of Pi content

The Pi content was measured following a modified protocol as previously described [80]. Plant tissues were ground into fine powder in liquid nitrogen. Approximately 0.1 g of tissues was sampled into 1.5 mL EP tubes. The samples were completely suspended and homogenized in extraction buffer [10 mM Tris-HCl, 1 mM EDTA, 10 mM NaCl, 1 mM Beta-ME, and 1 mM PMSF, pH 8.0] and kept at room temperature for at least 30 min. The homogenate (100 μL) was separated into three new 1.5-mL EP tubes and 900 μL of 1% acetic acid was added to each tube and mixed thoroughly. The mixture was centrifuged at 13,000 rpm for 5 min, and then 300 μL of supernatant was separated into a new 1.5-mL EP tube. Thereafter, 700 μL of analysis buffer [0.35% (NH_4_)_2_MoO_4_, 2.34% H_2_SO_4_, and 1.4% VC] was added to each sample and kept at 42°C for 30 min. The Pi content was determined by measuring the absorbance of A_820_. The Pi concentration was computed by normalizing the A_820_ values to the initial weight (0.1 g), and the obtained values were subjected to data analysis.

### 4.10 RNA-seq analysis

The roots from *zmarf1* knockout mutant and WT seedlings were sampled under normal growth conditions. Each genotype contained ten plants with similar growth and each genotype was separated into two biological replicates for RNA-seq analysis. Total RNA was extracted and purified as previously described. RNA-seq libraries were constructed and sequenced on an Illumina NovaSeq 6000 platform and 150 bp paired-end reads were generated according to the standard protocol provided by OE Biotech (Shanghai, China). High-quality reads were aligned to the maize reference genome sequence (B73 Genome v4.0) using HISAT2 software (version 2.2.1.0). The FPKM of each gene was calculated, and the read counts of each gene were obtained using HTSeq-count. DESeq was used for differential gene expression analysis. A Q value < 0.01 and fold-change > 2, or fold-change < 0.5 was set as the threshold for significantly differential expression genes (DEGs). GO (http://geneontology.org/) enrichment of the DEGs was implemented using a hypergeometric test, in which the p-value was calculated and adjusted to the q-value. GO terms (*p*< 0.01 were considered significantly enriched. Integrative Genomics Viewer (http://software.broadinstitute.org/software/igv) was used to visualize the reads of the selected genes.

### 4.11 Yeast one-hybrid (Y1H) assay

Y1H assays were conducted using the Matchmaker Gold Yeast One Hybrid Screening System (Clontech, USA). The 2.5-kb promoter region of *ZmLBD1* or a 2-kb promoter region of *ZmARF1* was PCR-amplified and cloned into the pABAi vector at the *Sac* I and *Xho* I sites. The pABAi:proZmLBD1 and pABAi:proZmARF1 bait constructs were linearized using the *BstbI* restriction enzyme. The pABAi:proZmLBD1 construct was transformed into the Y1H Gold yeast strain together with the AD-ZmARF1 prey vector. Positive transformants were selected on SD/-L /-U medium containing aureobasidin (AbA) (Clontech, USA). The primers used are listed in S1 Table.

pABAi:proZmARF1 was transformed into the Y1H Gold yeast strain together with the AD cDNA library plasmid, and screened on SD/-L /-U-containing aureobasidin (AbA). The primers used are listed in S1 Table. Single colonies were PCR-amplified, and the sequences annotated as transcription factors were selected. pABAi:proZmARF1 was transformed into the Y1H Gold yeast strain together with AD constructs of the selected candidate transcription factors and screened on SD/L/U medium containing aureobasidin (AbA). The primers used are listed in S1 Table.

### 4.12 Electrophoretic mobility shift assay (EMSA)

The full-length CDS of *ZmARF1* and *ZmERF2* were PCR-amplified and cloned into pMAL-c2x at the *Eco*R I / *Hin*d III sites. The resulting MBP-ZmARF1 and MBP-ZmERF2 fusion constructs and an empty MBP plasmid were used to transform *Escherichia coli* strain DE3 (TransGen, Beijing, China). Expression of MBP-ZmARF1, MBP-ZmERF2 and MBP was induced by 0.2 mM isopropyl-β-d-1-thiogalactopyranoside (IPTG), and purified using amylose resin (NEB, E8021S#) according to the manufacturer’s instructions. We synthesized a 5’ biotin labeled probes of GC box and ABRE motifs, together with their respective unlabeled and mutated competitors (Sangon Biotech, Shanghai, China). All sequences are indicated in S1 Table. Downstream EMSA was performed using the MBP-ZmARF1 recombinant protein, a biotin-labeled GC box probe, an unlabeled GC box probe, and its mutated competitors. An upstream EMSA was performed using the MBP-ZmERF2 recombinant protein, a biotin-labeled ABRE motif probe, an unlabeled ABRE motif probe, and its mutated competitors.

All EMSA experiments were performed using a LightShift Chemiluminescent EMSA Kit (Thermo Fisher Scientific, Prod# 89880) following the manufacturer’s instructions with slight modifications. The 10 μL EMSA binding reaction contained 7 uL purified MBP-ZmARF1 or MBP-ZmERF2 or MBP, 1 μL binding buffer and 2 μL 20 fmol biotin-labeled probe. For competition assays involving the unlabeled probe and mutant competitors, 10-μL EMSA binding reaction contained 5 uL purified MBP-ZmARF1 or MBP-ZmERF2, 1 μL binding buffer, 2 μL 20 fmol biotin-labeled probe, and 2 μL of either unlabeled or mutant competitor. The reactions were incubated at 25°C for 30 min, electrophoresed on a 7% polyacrylamide gel, and transferred to a nylon membrane (Millipore INYC00010#). Signals were visualized using an imaging system (E-Blot; Shanghai, China).

### 4.13 Dual-luciferase assay

The 2 kb promoter region of *ZmARF1* and 2.5 kb promoter region of *ZmLBD1*were amplified by PCR and cloned into pGreenII0800-LUC to obtain the Pro*ZmARF1*:LUC and Pro*ZmLBD1*:LUC reporter constructs. The full-length CDS of *ZmERF2* and *ZmARF1* were cloned into pGreenII62SK to construct effectors. pGreenII62SK-GFP cells were used as controls. ProZmARF1: LUC-and ProZmLBD1:LUC-reporter constructs and their respective effector constructs were expressed in *N*. *benthamiana* leaves as previously described [81]. Luciferase activity assay was performed using a dual luciferase reporter assay kit (Vazyme, Nanjing, China) according to the manufacturer’s protocol. Relative LUC activity was calculated as the ratio of LUC to Ren activity. Each sample was tested in triplicate. All primers used are listed in S1 Table.

## Supporting information

S1 Fig Genetic variation in *ZmARF1* are associated with low Pi stress traits in maize. SNPs) in *ZmARF1* have been identified in 356 inbred maize lines. Different SNP sites were associated with (A) and (B) plant height (PH), (C) and (D) total root tip (TRT) number, and (E) and (F) total root length (TRL). HP = high-Pi condition; LP = low-Pi condition. The statistical significance between SNPs was determined using a two-sided *t*-test.
(TIF)

S2 Fig Haplotypes of *ZmARF1* identified in the natural variation panel. Haplotypes of *ZmARF1* identified in 356 inbred maize lines associated with (A) and (B) plant height (PH), (C) and (D) total root tip (TRT) number, and (E) and (F) total root length (TRL) in high-Pi (HP) and low-Pi (LP) conditions, respectively. where n denotes the number of germplasm lines for each haplotype. The statistical significance between SNPs was determined using a two-sided *t*-test.
(TIF)

S3 Fig Overexpression of *ZmARF1* in the maize KN5585 background. **(A)** schematic representation of the construct used to overexpress *ZmARF1* in KN5585 cells. (B) Immunoblotting assay of transgenic maize revealing ZmARF1 expression levels in the respective overexpression lines.
(TIF)

S4 Fig CRISPR/Cas9-mediated knockout of ZmARF1 in maize. (A) Schematic representation of full-length ZmARF1 coding sequence and its conserved domains. The sgRNA targeted the 10^th^ exon located on the AUX/IAA functional domain at the C-terminus. (B) Alignment of the genomic sequences of zmarf1 mutants with sgRNA and WT reference sequences to identify the genotypes of independent mutant lines. The red arrows indicate the positions of the respective base pair(s) deletions. (C) RT-qPCR revealed the transcript abundance of ZmARF1 in WT and zmarf1 mutants. The transcript level of ZmARF1 was significantly reduced in the two zmarf1 mutants compared to the WT. The error bars represent the standard error of the mean of triplicate experiments.
(TIF)

S5 Fig Gene ontology enrichment of proteins encoded by differentially expressed genes. Functional assignment of DEGs by GO analysis; bar represents p value.
(TIF)

S6 Fig RNA-seq analysis of up regulated DEGs in knockout mutants of ZmARF1. (A) Venn diagram showing all identified DEGs in zmarf1 overlapped with RDMGs. (B) Overlap of upregulated DEGs in zmarf1 knockout mutants with RDMGs. Fisher’s exact test was used to calculate p-values. The scale represents the fold change. (C–F) Visualization of the RNA-seq coverage profile for (C) Zm0001d043179, (D) Zm00001d018751, (E) Zm00001d013245, and (F) Zm00001d018751 using the Integrated Genome Viewer browser. The scale represents the normalized read counts.
(TIF)

S7 Fig RT-qPCR analysis of the representative down regulated root development genes. Expression of *ZmLBD1* (Zm00001d027678), RTCS-like 1 (Zm00001d048401), putative auxin efflux carrier (Zm00001d044812), spermine synthase1 (Zm00001d046112), sucrose transport protein SUT1-like (Zm00001d048311), and LOB domain-containing protein 29 (Zm00001d031882). The expression level of each DEG in zmarf1 mutant was analyzed by RT-qPCR and normalized to ZmActin2/ZmGAPDH expression, and the relative expression ratio was calculated as 2^–ΔΔCt^ compared to that of WT. Three biological replicates were used, and three plants of each genotype were used for each replicate.
(TIF)

S8 Fig Activity detection of the different promoter regions of *ZmLBD1*. (A) A schematic diagram of five segmentations of *the ZmLBD1* promoter. (B) Detection of transcriptional activity of five fragments in *the ZmLBD1* promoter. Segments 3 and 5 (P3 and P5, respectively) exhibited strong transcriptional activity that was not inhibited by high concentrations of Aureobasidin A (AbA). An empty pABAi vector was used as control.
(TIF)

S9 Fig Genotype detection of EMS-induced mutant zmlbd1 in maize, and double mutant lbd16 lbd18 in Arabidopsis. (A) Schematic representation of EMS-induced mutation site in the exon of ZmLBD1 and alignment of genomic sequence of zmlbd1 mutants with B73, zmlbd1 and control reference sequence to identify genotypes of independent mutant lines (B) Genomic DNA extracted from Col-0, double mutant lbd16 lbd18 and lbd16 lbd18/35S::ZmLBD1 was subjected to PCR amplification with primers specific to LBD16 or LBD18 and the general primers for T-DNA. LP and RP represent the specific left and right primers, respectively, and LB represents the general primer. C1#, C2#, and C3# represent three independent complementary lines. M = DNA marker, 1–24 represents partly independent single clones.
(TIF)

S10 Fig Y1H library screening assay. (A) Y1H gold strains were transformed with the bait construct pABAi-Pro_ZmARF1_. (B) Positive yeast cells carrying the construct pABAi-Pro_ZmARF1_ were transformed with the cDNA library plasmid in pGADT7 and grown on SD/-U medium containing 100–300 ng mL^-1^ AbA. Single independent colonies were subjected to PCR amplification using common AD prey vector primers (T7-F/3AD-R). M = DNA marker, 1–24 represents partly independent single clones.
(TIF)

S1 Table Primers used in this study. (XLSX)

S2 Table SNP polymorphisms of ZmARF1 in 356 maize inbreds. (XLSX)

S3 Table Associations between the nucleotide polymorphisms in ZmARF1 and phosphorus-deficiency-tolerance traits in maize. (XLSX)

S4 Table Candidate genes identified from Y1H library screening. (XLSX)
