# Supplementary figures and images for: ZmARF1 positively regulates low phosphorus stress tolerance via modulating lateral root development in maize
Source: PLoS Genet. 2024 Feb 5;20(2):e1011135. doi: 10.1371/journal.pgen.1011135 (PMC10868794; doi:10.1371/journal.pgen.1011135)

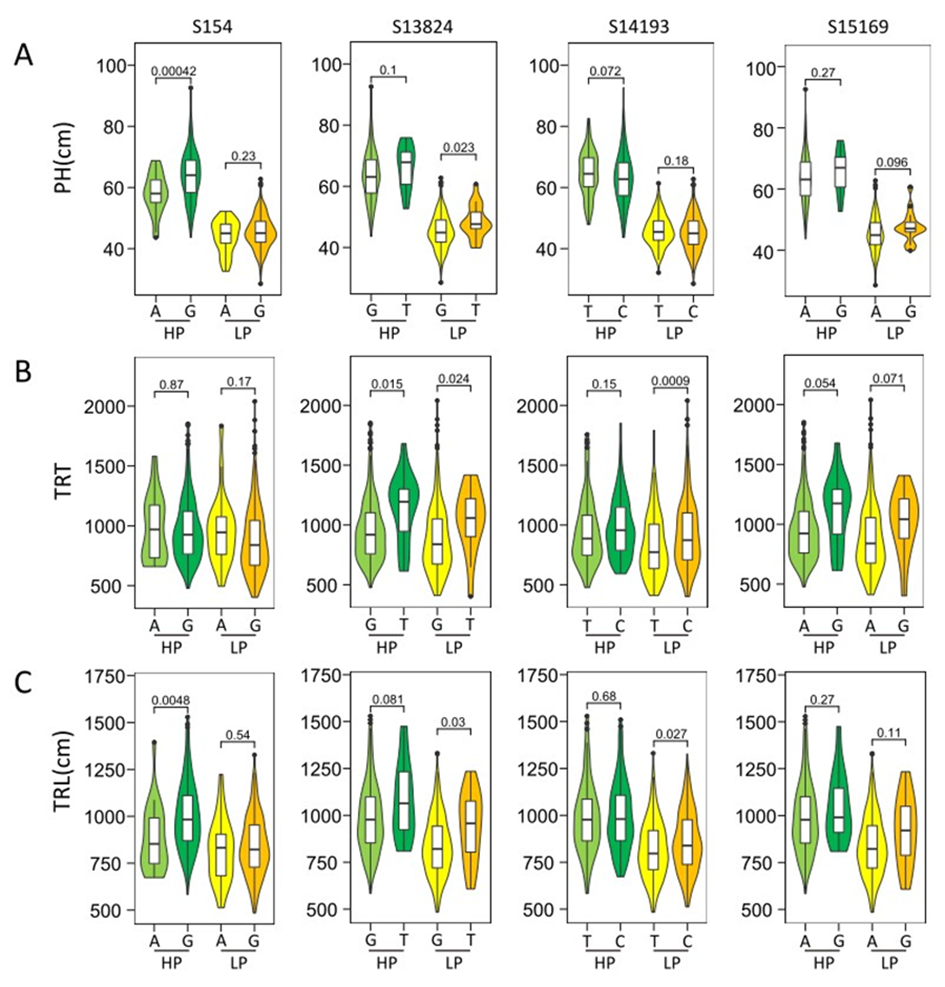

Supplement: S1 Fig — SNPs) in ZmARF1 have been identified in 356 inbred maize lines. Different SNP sites were associated with (A) and (B) plant height (PH), (C) and (D) total root tip (TRT) number, and (E) and (F) total root length (TRL). HP = high-Pi condition; LP = low-Pi condition. The statistical significance between SNPs was determined using a two-sided t-test. (TIF) [file pgen.1011135.s001.tif]

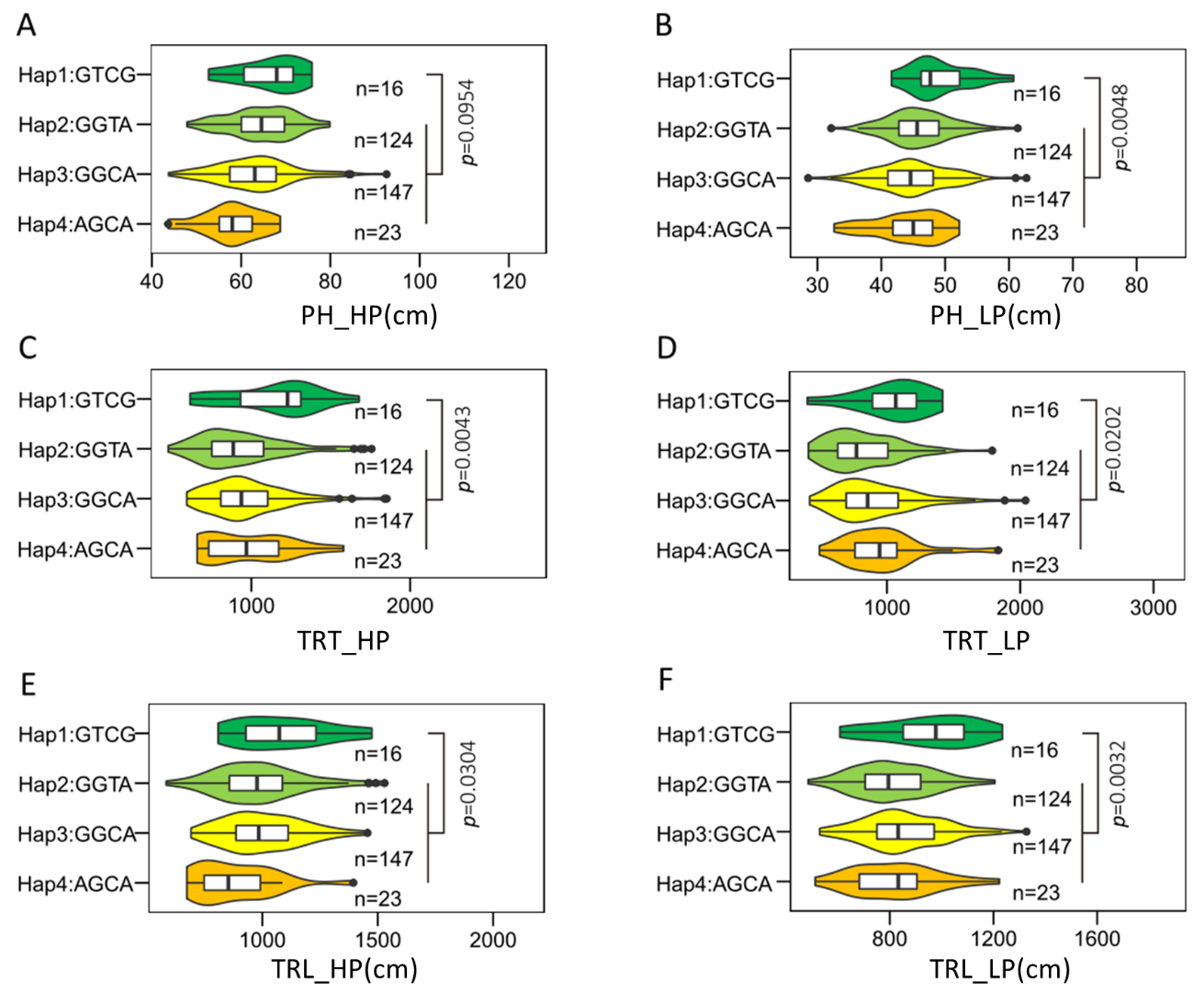

Supplement: S2 Fig — Haplotypes of ZmARF1 identified in 356 inbred maize lines associated with (A) and (B) plant height (PH), (C) and (D) total root tip (TRT) number, and (E) and (F) total root length (TRL) in high-Pi (HP) and low-Pi (LP) conditions, respectively. where n denotes the number of germplasm lines for each haplotype. The statistical significance between SNPs was determined using a two-sided t-test. (TIF) [file pgen.1011135.s002.tif]

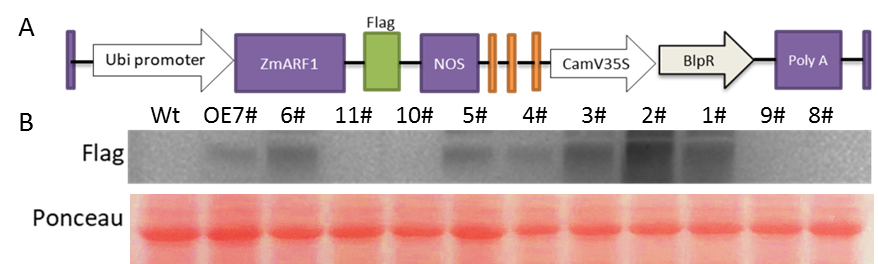

Supplement: S3 Fig — (A) schematic representation of the construct used to overexpress ZmARF1 in KN5585 cells. (B) Immunoblotting assay of transgenic maize revealing ZmARF1 expression levels in the respective overexpression lines. (TIF) [file pgen.1011135.s003.tif]

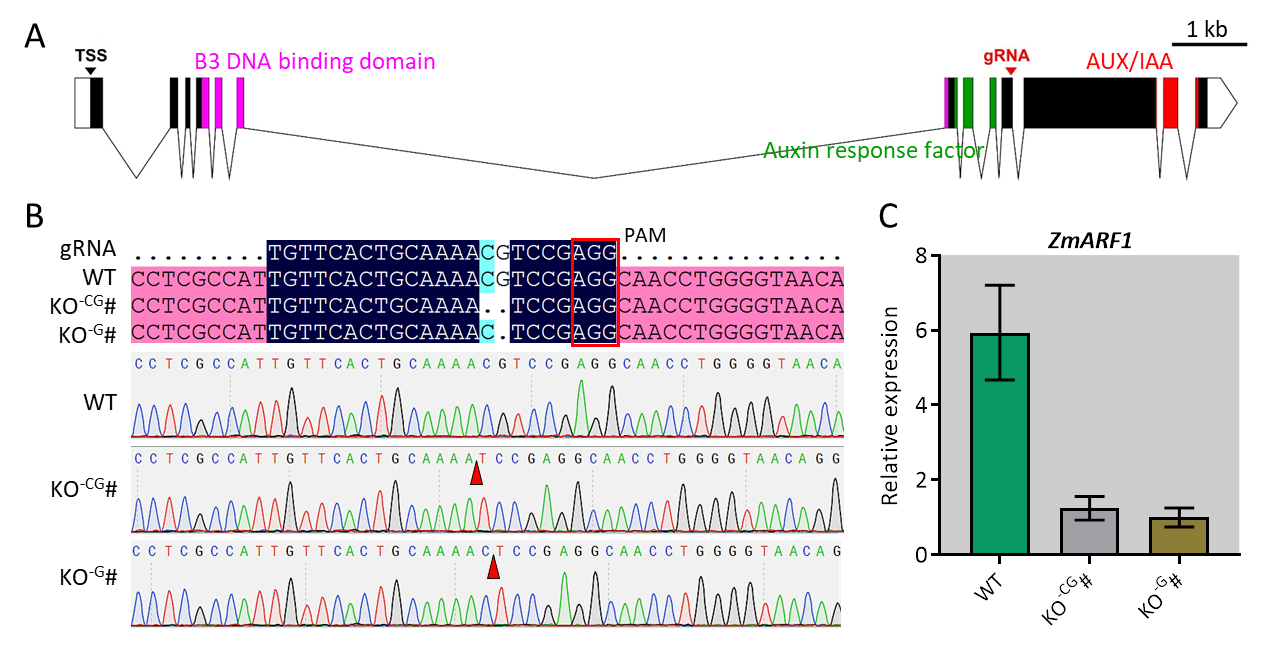

Supplement: S4 Fig — (A) Schematic representation of full-length ZmARF1 coding sequence and its conserved domains. The sgRNA targeted the 10th exon located on the AUX/IAA functional domain at the C-terminus. (B) Alignment of the genomic sequences of zmarf1 mutants with sgRNA and WT reference sequences to identify the genotypes of independent mutant lines. The red arrows indicate the positions of the respective base pair(s) deletions. (C) RT-qPCR revealed the transcript abundance of ZmARF1 in WT and zmarf1 mutants. The transcript level of ZmARF1 was significantly reduced in the two zmarf1 mutants compared to the WT. The error bars represent the standard error of the mean of triplicate experiments. (TIF) [file pgen.1011135.s004.tif]

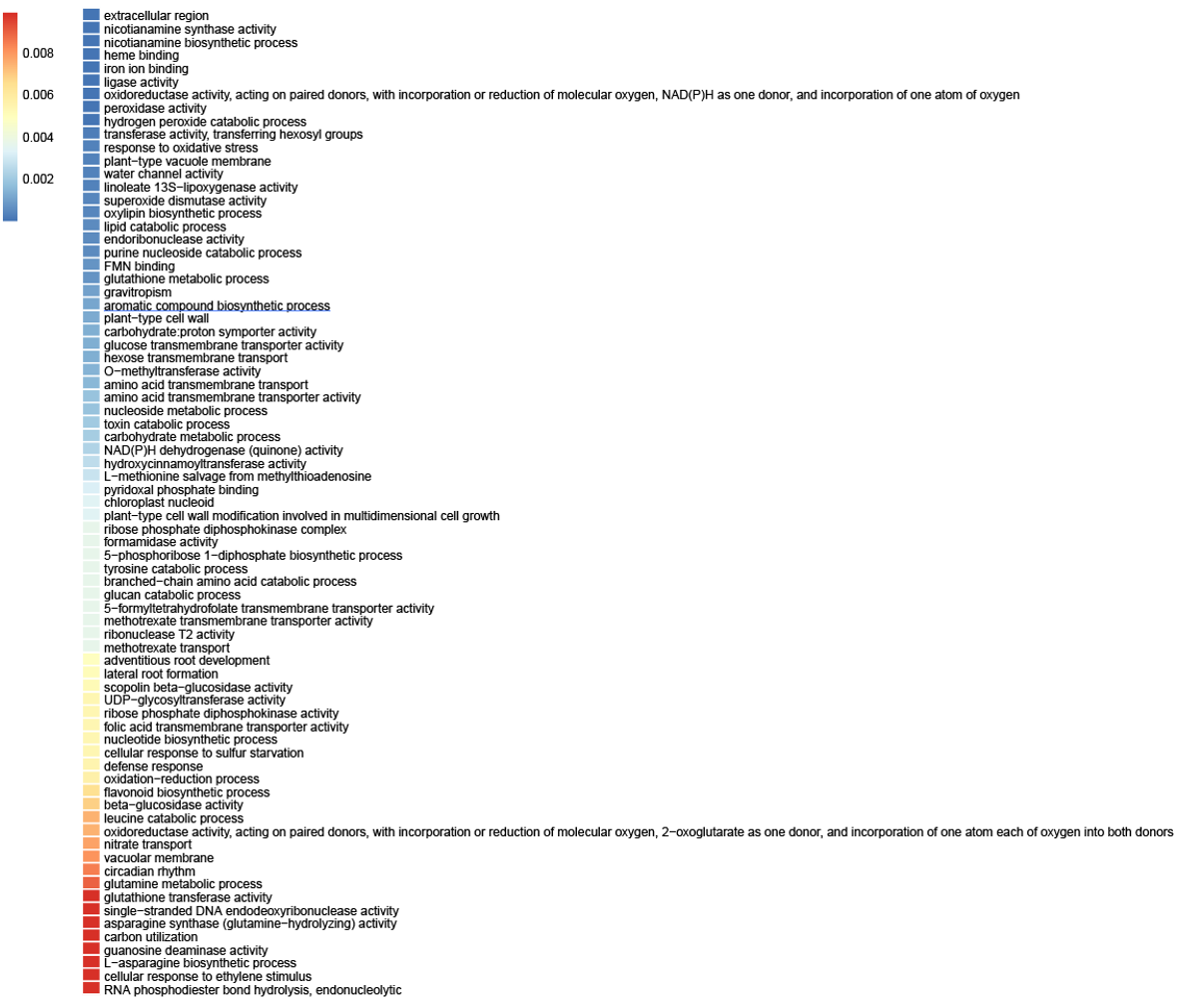

Supplement: S5 Fig — Functional assignment of DEGs by GO analysis; bar represents p value. (TIF) [file pgen.1011135.s005.tif]

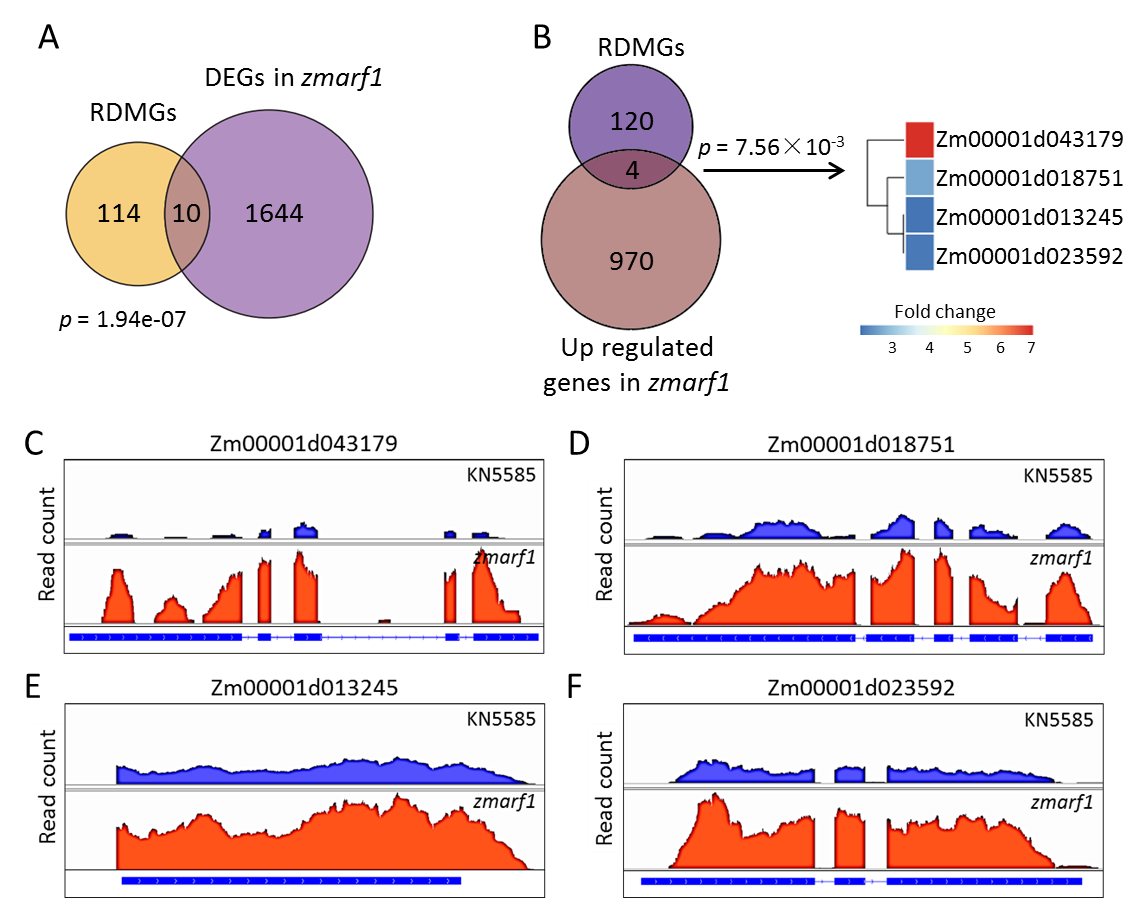

Supplement: S6 Fig — (A) Venn diagram showing all identified DEGs in zmarf1 overlapped with RDMGs. (B) Overlap of upregulated DEGs in zmarf1 knockout mutants with RDMGs. Fisher’s exact test was used to calculate p-values. The scale represents the fold change. (C–F) Visualization of the RNA-seq coverage profile for (C) Zm0001d043179, (D) Zm00001d018751, (E) Zm00001d013245, and (F) Zm00001d018751 using the Integrated Genome Viewer browser. The scale represents the normalized read counts. (TIF) [file pgen.1011135.s006.tif]

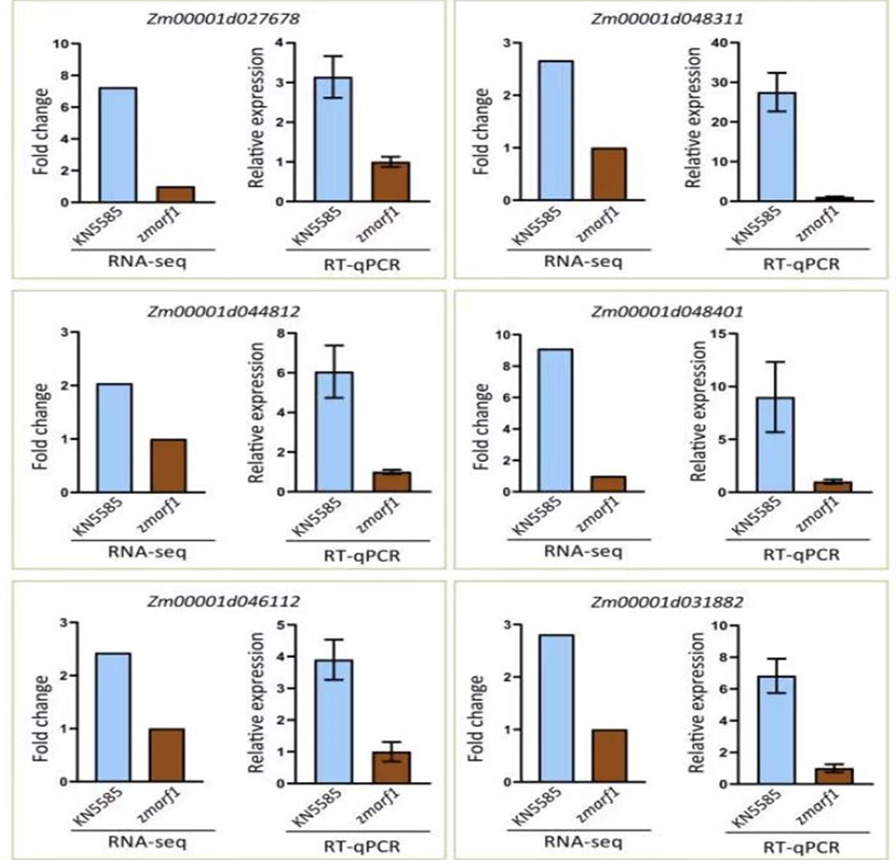

Supplement: S7 Fig — Expression of ZmLBD1 (Zm00001d027678), RTCS-like 1 (Zm00001d048401), putative auxin efflux carrier (Zm00001d044812), spermine synthase1 (Zm00001d046112), sucrose transport protein SUT1-like (Zm00001d048311), and LOB domain-containing protein 29 (Zm00001d031882). The expression level of each DEG in zmarf1 mutant was analyzed by RT-qPCR and normalized to ZmActin2/ZmGAPDH expression, and the relative expression ratio was calculated as 2–ΔΔCt compared to that of WT. Three biological replicates were used, and three plants of each genotype were used for each replicate. (TIF) [file pgen.1011135.s007.tif]

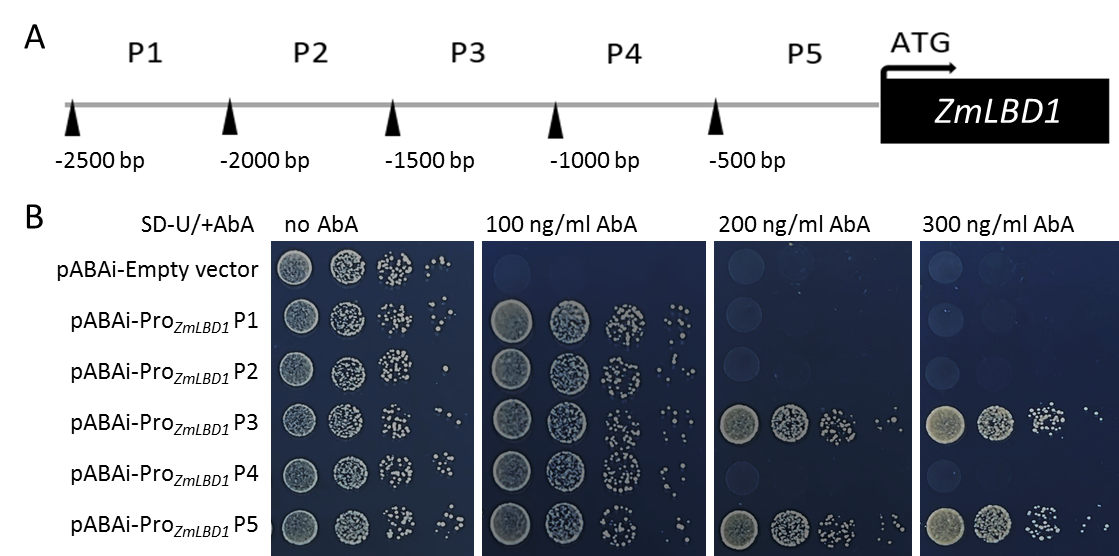

Supplement: S8 Fig — (A) A schematic diagram of five segmentations of the ZmLBD1 promoter. (B) Detection of transcriptional activity of five fragments in the ZmLBD1 promoter. Segments 3 and 5 (P3 and P5, respectively) exhibited strong transcriptional activity that was not inhibited by high concentrations of Aureobasidin A (AbA). An empty pABAi vector was used as control. (TIF) [file pgen.1011135.s008.tif]

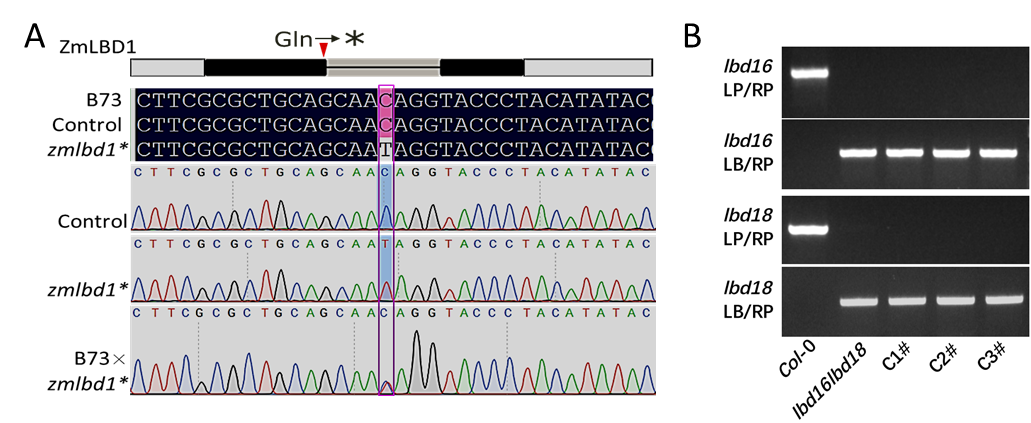

Supplement: S9 Fig — (A) Schematic representation of EMS-induced mutation site in the exon of ZmLBD1 and alignment of genomic sequence of zmlbd1 mutants with B73, zmlbd1 and control reference sequence to identify genotypes of independent mutant lines (B) Genomic DNA extracted from Col-0, double mutant lbd16 lbd18 and lbd16 lbd18/35S::ZmLBD1 was subjected to PCR amplification with primers specific to LBD16 or LBD18 and the general primers for T-DNA. LP and RP represent the specific left and right primers, respectively, and LB represents the general primer. C1#, C2#, and C3# represent three independent complementary lines. M = DNA marker, 1–24 represents partly independent single clones. (TIF) [file pgen.1011135.s009.tif]

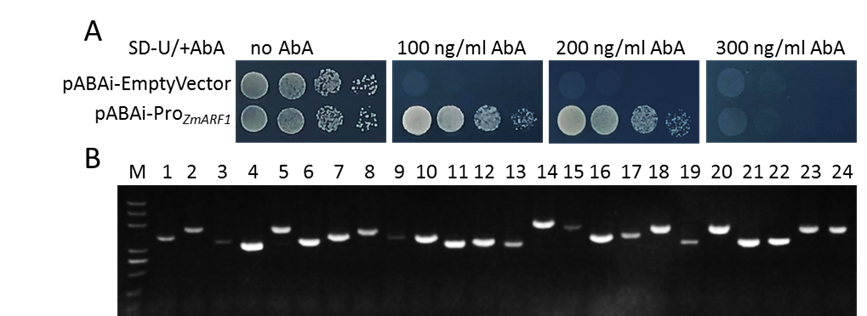

Supplement: S10 Fig — (A) Y1H gold strains were transformed with the bait construct pABAi-ProZmARF1. (B) Positive yeast cells carrying the construct pABAi-ProZmARF1 were transformed with the cDNA library plasmid in pGADT7 and grown on SD/-U medium containing 100–300 ng mL-1 AbA. Single independent colonies were subjected to PCR amplification using common AD prey vector primers (T7-F/3AD-R). M = DNA marker, 1–24 represents partly independent single clones. (TIF) [file pgen.1011135.s010.tif]
